# A fluid biomarker reveals loss of TDP-43 splicing repression in presymptomatic ALS–FTD

**DOI:** 10.1038/s41591-023-02788-5

**Published:** 2024-01-26

**Authors:** Katherine E. Irwin, Pei Jasin, Kerstin E. Braunstein, Irika R. Sinha, Mark A. Garret, Kyra D. Bowden, Koping Chang, Juan C. Troncoso, Abhay Moghekar, Esther S. Oh, Denitza Raitcheva, Dan Bartlett, Timothy Miller, James D. Berry, Bryan J. Traynor, Jonathan P. Ling, Philip C. Wong

**Affiliations:** 1 Department of Pathology, Johns Hopkins Medicine, Baltimore, MD USA; 2 Department of Neuroscience, Johns Hopkins Medicine, Baltimore, MD USA; 3 Sean M. Healey & AMG Center for ALS, Massachusetts General Hospital, Boston, MA USA; 4 Department and Graduate Institute of Pathology, National Taiwan University Hospital, National Taiwan University College of Medicine, Taipei, Taiwan; 5 Department of Neurology, Johns Hopkins Medicine, Baltimore, MD USA; 6 Department of Medicine, Johns Hopkins Medicine, Baltimore, MD USA; 7 Department of Psychiatry and Behavioral Sciences, Johns Hopkins Medicine, Baltimore, MD USA; 8 Biogen, Cambridge, MA USA; 9 Department of Neurology, Washington University School of Medicine in St. Louis, St. Louis, MO USA; 10 Neuromuscular Diseases Research Section, National Institute on Aging, National Institutes of Health, Bethesda, MD USA; 11 National Institute of Neurological Disorders, National Institutes of Health, Bethesda, MD USA; 12 RNA Therapeutics Laboratory, Therapeutics Development Branch, National Center for Advancing Translational Sciences, National Institutes of Health, Rockville, MD USA

**Keywords:** Amyotrophic lateral sclerosis, Diagnostic markers

## Abstract

Although loss of TAR DNA-binding protein 43 kDa (TDP-43) splicing repression is well documented in postmortem tissues of amyotrophic lateral sclerosis (ALS) and frontotemporal dementia (FTD), whether this abnormality occurs during early-stage disease remains unresolved. Cryptic exon inclusion reflects loss of function of TDP-43, and thus detection of proteins containing cryptic exon-encoded neoepitopes in cerebrospinal fluid (CSF) or blood could reveal the earliest stages of TDP-43 dysregulation in patients. Here we use a newly characterized monoclonal antibody specific to a TDP-43-dependent cryptic epitope (encoded by the cryptic exon found in *HDGFL2*) to show that loss of TDP-43 splicing repression occurs in ALS–FTD, including in presymptomatic *C9orf72* mutation carriers. Cryptic hepatoma-derived growth factor-like protein 2 (HDGFL2) accumulates in CSF at significantly higher levels in familial ALS–FTD and sporadic ALS compared with controls and is elevated earlier than neurofilament light and phosphorylated neurofilament heavy chain protein levels in familial disease. Cryptic HDGFL2 can also be detected in blood of individuals with ALS–FTD, including in presymptomatic *C9orf72* mutation carriers, and accumulates at levels highly correlated with those in CSF. Our findings indicate that loss of TDP-43 cryptic splicing repression occurs early in disease progression, even presymptomatically, and that detection of the HDGFL2 cryptic neoepitope serves as a potential diagnostic biomarker for ALS, which should facilitate patient recruitment and measurement of target engagement in clinical trials.

## Abstract (web summary)

This study identifies a fluid biomarker of TDP-43 dysfunction, a central pathological feature of the ALS–FTD disease spectrum, and demonstrates that such loss of TDP-43 splicing repression occurs presymptomatically.

## Main

A fluid biomarker for the presymptomatic or prodromal phases of ALS–FTD to enable earlier diagnosis, and to facilitate patient recruitment and monitor target engagement in clinical trials, is a great unmet need. A central pathological hallmark of the ALS–FTD disease spectrum is the nuclear mislocalization and cytoplasmic aggregation of DNA/RNA-binding protein TDP-43 (ref. ^1^). While a gain-of-function mechanism due to TDP-43 cytoplasmic aggregates has been proposed to contribute to neurodegeneration^2–6^, emerging evidence supports the idea that loss of TDP-43 repression of cryptic splicing resulting from depletion of nuclear TDP-43 drives neuron loss in ALS–FTD^7,8^. Because TDP-43 pathology can currently be revealed only with postmortem analysis, while such TDP-43 functional deficits are well documented in end-stage tissues^9–15^, the extent to which loss of TDP-43 splicing repression occurs during the early stages of disease is unclear. Clarification of this question would provide critical insight into disease mechanisms and inform therapeutic strategies designed to attenuate neuron loss in ALS–FTD.

**Extended Data Fig. 1:**
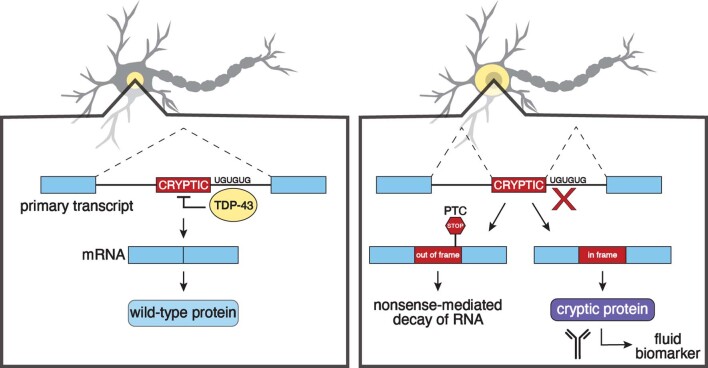
Strategy for developing cryptic peptide fluid biomarkers. TDP-43 normally binds to UG repeats flanking cryptic exons and prevents them from being incorporated into messenger RNA (mRNA). When TDP-43 is lost from the nucleus, it fails to repress the splicing of cryptic exons. As some cryptic exons are incorporated in-frame, antibodies can be developed against cryptic exon-encoded peptides to serve as fluid biomarkers. PTC: premature termination codon.

Loss of TDP-43 splicing repression leads to the inclusion of numerous nonconserved cryptic exons, of which about 3% produce in-frame neoepitopes^7,16^. We hypothesize that detection of cryptic exon-encoded peptides in biofluids could reveal how early TDP-43 splicing repression is dysregulated in patients with ALS–FTD and could establish fluid biomarkers that reflect TDP-43 dysfunction (Extended Data Fig. 1). To test this idea we selected certain cryptic neoepitopes for antibody generation based on RNA expression data and protein structure modeling. We then validated these novel monoclonal antibodies in HeLa cells depleted of TDP-43 by small interfering RNA. We focus here on one antibody that reliably detected a cryptic exon-encoded neoepitope in HDGFL2. Using this novel antibody we developed a highly specific and sensitive sandwich ELISA to determine the dynamic nature of this cryptic exon-encoded neoepitope in CSF from individuals with sporadic ALS, as well as in CSF and blood from presymptomatic and symptomatic individuals with *C9orf72* mutations causing familial ALS–FTD^17,18^.

## Results

### Selection of TDP-43-dependent cryptic exon targets

**Fig. 1:**
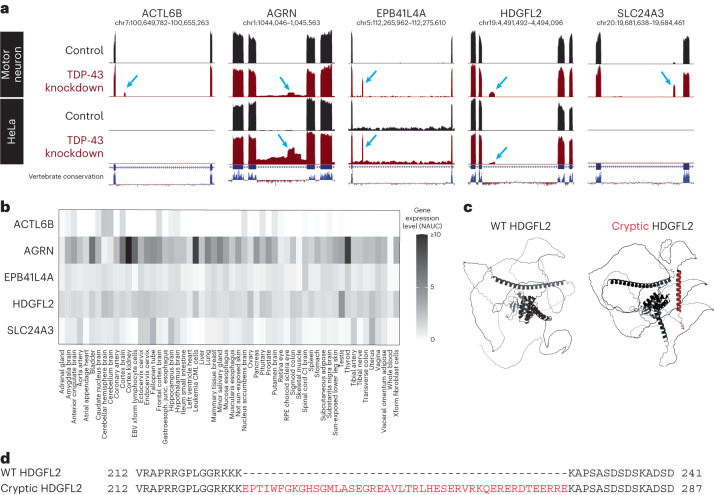
Identification of human in-frame, TDP-43-associated cryptic exons. **a**, UCSC Genome Browser visualization of selected cryptic exons in human motor neurons^11^ and HeLa cells^7^ aligned to the GRCh38 assembly. Red tracks indicate TDP-43 knockdown, and blue arrows identify nonconserved cryptic exons. Gene annotations below RNA sequencing tracks indicate canonical exons (thick lines) and introns (thin lines). **b**, Visualization of tissue type-specific gene expression of *ACTL6B*, *AGRN*, *EPB41L4A*, *HDGFL2* and *SLC24A3*. While *AGRN* and *HDGFL2* RNA transcripts are ubiquitously expressed, *ACTL6B*, *EPB41L4A* and *SLC24A3* are expressed in a more tissue-specific manner. NAUC values derived from ASCOT^19^ are used to approximate gene expression levels in different human tissue types. **c**, Comparison of WT (left) and cryptic (right) HDGFL2 protein structures. Inclusion of the cryptic exon in mRNA leads to the addition of 46 amino acids predicted to form an alpha-helix structure (red) between flanking unstructured regions. Both structures are generated using AlphaFold predictions derived from amino acid sequences. The WT HDGFL2 protein structure can be found on the AlphaFold protein structure database (UniProt: Q7Z4V5). **d**, Alignment of WT and cryptic HDGFL2 amino acid sequences. The cryptic inclusion is 46 amino acids long (red) and does not impact flanking amino acids. Source data

A series of human TDP-43-associated cryptic exons were identified from RNA sequencing of HeLa cells^7^ and induced pluripotent stem cell-derived motor neurons^11,13^ depleted of TDP-43 using small interfering RNA (siRNA). Some of these cryptic exons were selected as targets (Fig. 1a) for the development of monoclonal antibodies based on the following criteria: (1) the cryptic exon is spliced in frame without a premature termination codon; (2) the cryptic exon-containing gene is ubiquitously expressed or expressed abundantly in the central nervous system (CNS); and (3) the cryptic exon-encoded peptide contains immunogenic epitopes.

Genes harboring TDP-43-related cryptic exons were analyzed using an alternative splicing catalog of the transcriptome (ASCOT) generated using publicly available bulk RNA sequencing datasets^19^. In-frame cryptic exons in genes with high expression across different human tissues were selected for broad relevance to TDP-43-related diseases. In-frame cryptic exons in genes with high expression in the CNS were also selected due to their expected involvement in ALS–FTD (Fig. 1b). Following selection of cryptic exon targets based on the initial two criteria, AlphaFold protein structure prediction software^20^ was used to model the cryptic peptide-containing proteins of interest to visualize the accessibility of cryptic epitopes and predict the preservation of proteins’ native conformations (Fig. 1c and Supplementary Fig. 1). Cryptic exon-encoded epitopes with immunogenic potential that did not substantially disrupt protein conformation or interfere with binding of antibodies targeting wild-type (WT) sequences were selected as prospective targets.

Of the cryptic exons meeting these criteria (Fig. 1 and Supplementary Fig. 1), one promising target selected for development of novel monoclonal antibodies was the cryptic exon-encoded epitope within hepatoma-derived growth factor-like protein 2 (HDGFL2), a histone-binding protein that is nearly ubiquitously expressed and is detected in brain and spinal motor neurons (Fig. 1b). To generate antibodies against cryptic HDGFL2, mice were immunized with the *HDGFL2* cryptic exon-encoded peptide (Fig. 1d), antibody-secreting hybridoma cells were isolated for this target and antibodies were purified from the hybridoma cell lines.

### Novel antibody specific to cryptic neoepitope in HDGFL2

A three-part screening approach was used to evaluate the sensitivity and specificity of the novel monoclonal antibodies. We first screened for monoclonal lines that would recognize the cryptic exon-encoded peptide in HDGFL2 when expressed as a myc-tagged green fluorescent protein (GFP)–cryptic peptide fusion protein. Lysates from HEK293 cells transfected with either the GFP–myc-cryptic HDGFL2 fusion or GFP alone were subjected to protein blot analysis with monoclonal antibodies. Among monoclonal line nos. 1-65 to 1-71 against the cryptic HDGFL2 epitope, line nos. 1-66 and 1-69 detected with specificity the fusion protein containing the cryptic HDGFL2 peptide (Supplementary Fig. 2). Line no. 1-69, termed TC1HDG, was used moving forward.

**Fig. 2:**
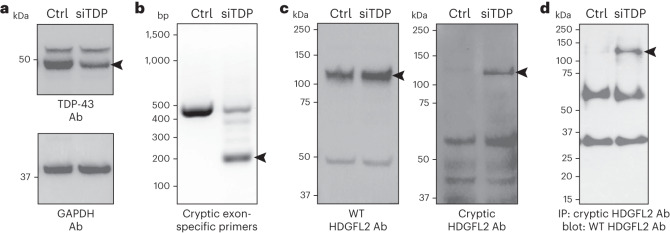
Novel antibody shows specificity for HDGFL2 with cryptic peptide. **a**, TDP-43 is reduced (arrowhead) in HeLa treated with TDP-43 siRNA (siTDP) compared with nontransfected control HeLa (ctrl). Ab, antibody. **b**, Reverse transcription PCR using primers designed to amplify the cryptic exon sequence of HDGFL2 shows a product (arrowhead) found only in siTDP. **c**, Protein extracts as in **a** were subjected to protein blot analysis using either an antibody (rabbit polyclonal antibody against human CTB-50L17.10, HPA044208) against the native HDGFL2 protein (left) or the novel monoclonal antibody (TC1HDG) against the cryptic sequence in HDGFL2 (right). While WT HDGFL2 was detected in both control and siTDP lysates (left and right bands at level of left arrowhead), HDGFL2 harboring the neoepitope (right arrowhead) was detected only in siTDP. **d**, IP blot using TC1HDG cryptic antibody for pulldown and WT HDGFL2 antibody for blotting revealed a band of the expected size only in siTDP (arrowhead). Lower bands represent presumable IgG heavy and light chains.

Second, we used an siRNA knockdown strategy in HeLa cells to deplete TDP-43 (siTDP HeLa) and screened antibodies in siTDP versus control (nontransfected) HeLa lysates (Fig. 2a). We confirmed, as expected, the appearance of *HDGFL2* containing a cryptic exon in siTDP as compared with control HeLa (Fig. 2b). We then subjected total cell extracts from control and siTDP HeLa cells to protein blot analysis with the monoclonal TC1HDG antibody to determine its specificity. As expected, a band corresponding to the molecular weight of the normal HDGFL2 protein was observed in both control and siTDP HeLa cells using an antibody recognizing the native HDGFL2 (Fig. 2c). Importantly, the TC1HDG cryptic antibody recognized a novel band of the expected size, presumably corresponding to the cryptic peptide-containing HDGFL2 (termed cryptic HDGFL2), in an extract of HeLa cells depleted of TDP-43 but not control cells (Fig. 2c). To further demonstrate the specificity of the monoclonal antibody we performed immunoprecipitation (IP) using TC1HDG followed by protein blot analysis with the antibody recognizing the native HDGFL2. While no positive band of correct molecular weight was observed in control HeLa, a robust band of expected size for the cryptic exon-encoded peptide within HDGFL2 was evident in siTDP HeLa cells (Fig. 2d). These data therefore demonstrate that the TC1HDG antibody specifically recognizes the cryptic exon-encoded peptide within HDGFL2.

### TC1HDG antibody detects cryptic neoepitope in ALS–FTD brain

**Fig. 3:**
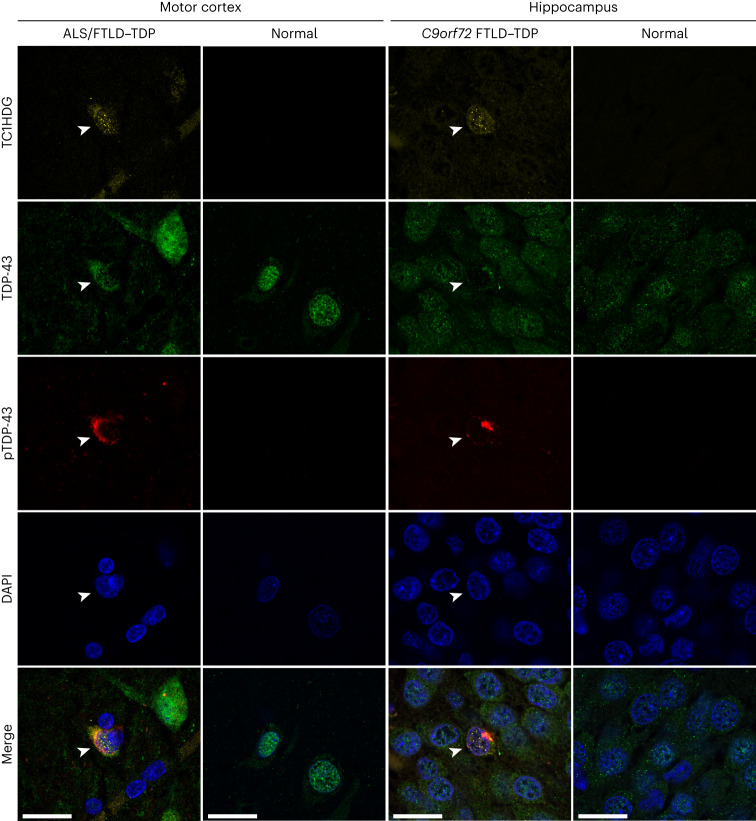
TC1HDG antibody detects cryptic HDGFL2 in neurons of the ALS–FTD brain. Cryptic HDGFL2 was detected by our TC1HDG cryptic antibody (first row, yellow) specifically in neurons (arrowheads) of the motor cortex (first column) or hippocampus (third column) that are depleted of nuclear TDP-43 (second row, green) and contain phosphorylated TDP-43 (pTDP-43) cytoplasmic aggregates (third row, red). Note that cryptic HDGFL2 immunoreactivity is largely restricted to the nuclear compartments. Neurons with intact nuclear TDP-43 did not show cryptic HDGFL2 immunoreactivity. Scale bars, 20 µm.

To determine whether cryptic HDGFL2 accumulates in neurons exhibiting mislocalization of TDP-43, immunocytochemical analysis using the TC1HDG antibody was performed in ALS–frontotemporal lobar degeneration (FTLD) and control brain tissue (Supplementary Table 1). In both ALS/FTLD–TDP motor cortex and *C9orf72*-linked FTLD–TDP hippocampus, TC1HDG immunoreactivity was seen specifically in nuclei of neurons displaying nuclear clearance of TDP-43 accompanied by phosphorylated TDP-43 cytoplasmic aggregates (Fig. 3). These results support the idea that cryptic *HDGFL2* messenger RNA can be translated in cells in which TDP-43 has been depleted from the nucleus, and also that cryptic HDGFL2 accumulates in neurons of the ALS–FTD brain.

### A highly sensitive ELISA for detection of cryptic HDGFL2

**Fig. 4:**
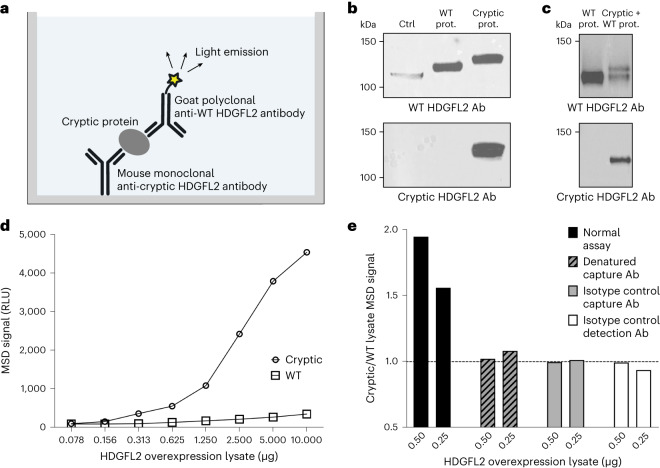
Development of an MSD assay specific for cryptic HDGFL2. **a**, Sandwich ELISA using the MSD system. **b**, A band corresponding to WT HDGFL2 was detectable by gTEA1.2 in lysates of nontransfected HeLa (ctrl), HeLa transfected to overexpress WT HDGFL2 (WT prot.) and HeLa transfected to overexpress cryptic HDGFL2 (cryptic prot.). A band corresponding to cryptic HDGFL2 was detectable by the TC1HDG cryptic antibody only in HeLa transfected to overexpress cryptic HDGFL2. **c**, A double band of the expected sizes was seen in HeLa lysate cotransfected to overexpress both cryptic and WT HDGFL2 (cryptic + WT prot.) when probed with an antibody against WT HDGFL2 (gTEA1.2). Probing with the TC1HDG cryptic antibody revealed a single band of the expected size in only the cryptic and WT co-overexpression lysate. **d**, A dose-dependent increase of MSD signal in the lysate of HeLa overexpressing cryptic HDGFL2 (circles) was observed compared with HeLa lysate overexpressing WT HDGFL2 (squares). **e**, Elevated MSD signal of cryptic HDGFL2 overexpression lysate is specific to intact capture and detection antibodies. Denatured capture antibody was heated at 95 °C for 30 min, and mouse IgG and sulfo-tagged goat IgG were used as isotype controls for capture and detection antibodies, respectively. RLU, relative light units.

To employ our TC1HDG antibody for detection of cryptic exon-encoded peptides in human CSF, we developed a highly sensitive sandwich ELISA using the Meso Scale Discovery (MSD) platform and validated this assay for the cryptic exon-encoded neoepitope in HDGFL2. In the same manner that we performed IP–protein blot analysis, we used the TC1HDG cryptic monoclonal antibody as the capture antibody to pull down HDGFL2 harboring the cryptic exon-encoded peptide and an antibody recognizing a WT HDGFL2 sequence as the detection antibody (Fig. 4a). We generated a goat polyclonal antibody (termed gTEA1.2), recognizing the same immunogen (Fig. 4b,c) as the previously used rabbit antibody against WT HDGFL2 (Fig. 2c,d), and conjugated this goat antibody with a sulfo-tag required for generation of quantifiable electrochemiluminescent signal.

We tested this MSD ELISA protocol using lysates of HeLa cells transfected to overexpress either cryptic or WT HDGFL2 (Fig. 4b). Compared with WT HDGFL2 overexpression lysate, a concentration-dependent increase in MSD signal was observed in cryptic HDGFL2 overexpression lysate (Fig. 4d). When the cryptic HDGFL2 capture antibody was denatured by heating at 95 °C for 30 min before addition to the plate, the ratio of cryptic:WT HDGFL2 lysate MSD signal decreased to the control level of 1, indicating there was no difference between MSD signals of cryptic and WT HDGFL2 overexpression lysates (Fig. 4e). In addition, when the cryptic HDGFL2 capture antibody was replaced with an isotype control antibody (mouse IgG) the ratio of cryptic:WT HDGFL2 decreased to 1. These data demonstrate the specificity of the assay for cryptic HDGFL2 binding by the capture antibody. When the detection antibody was replaced with a sulfo-tagged isotype control antibody (goat IgG), there was again no difference between MSD signals of cryptic and WT HDGFL2 overexpression lysates (Fig. 4e). When these antibody controls were employed for MSD measurement of a *C9orf72* CSF sample, the positive CSF signal decreased to below the level of diluent-only signal (Supplementary Fig. 3).

To further evaluate our assay we calculated both intra- and inter-assay coefficients of variation (CV). Intra-assay CV was determined to be 4.9% from 78 CSF samples measured in duplicate oriented on opposite ends of the MSD ELISA plate about an *x* and a *y* axis (39 duplicates on each of two plates). To determine inter-assay CV we measured ten CSF samples for cryptic HDGFL2 on two different MSD plates on different days. These ten CSF samples were derived from ten different *C9orf72* mutation carriers from whom at least two never-before-thawed CSF aliquots of the same CSF collection were available; the inter-assay CV for these samples was 2.9%. Taken together, these data establish a highly sensitive and specific sandwich ELISA for the detection of cryptic peptide-containing protein, a valuable method to monitor TDP-43 loss of function in biofluids of patients with ALS–FTD.

### Cryptic HDGFL2 is detectable in presymptomatic *C9orf72* CSF

**Extended Data Fig. 2:**
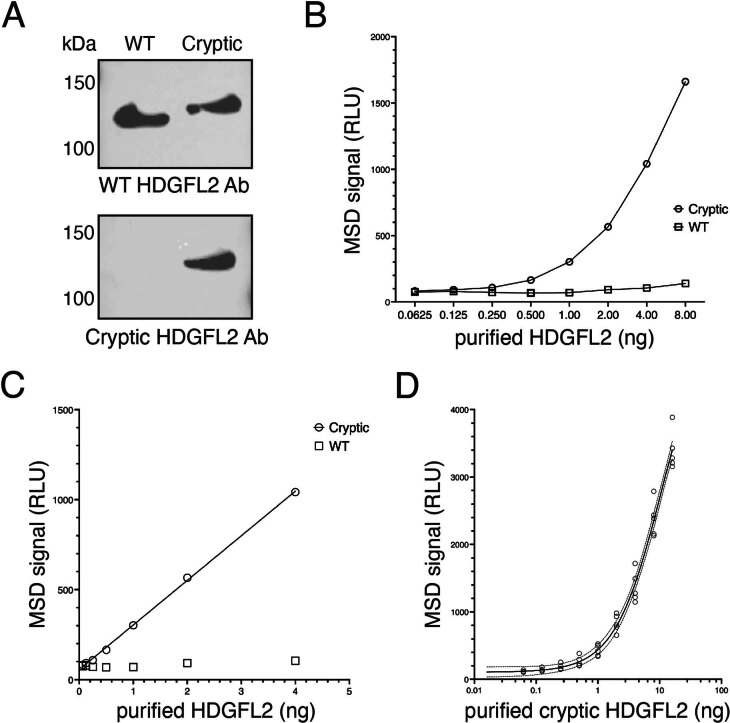
Standard curves used for quantification of cryptic HDGFL2. (**A**) Cryptic and WT HDGFL2 proteins were purified. Bands of the expected sizes are detectable by a goat antibody against WT HDGFL2 (gTEA1.2) for both proteins (upper panel). Only cryptic HDGFL2 shows a band of the expected size when probed with our novel TC1HDG cryptic antibody (lower panel). (**B**) There is a dose-dependent increase in MSD signal of purified cryptic HDGFL2 (circles) compared to WT HDGFL2 (squares). (**C**) At lower concentrations, MSD signal for purified cryptic HDGFL2 falls in a linear range (circles). MSD signal for WT HDGFL2 shown in squares. (**D**) Five standard curves from different MSD plates are shown and fitted with a four-parameter logistic (4PL) curve used for calculation of cryptic HDGFL2 concentration. Solid line represents the mean, and dotted lines represent the 95% confidence interval. R^2^ = 0.98. RLU: relative light units.

To quantify cryptic HDGFL2 in patient biofluids we developed a standard curve of known concentrations of purified cryptic HDGFL2, which was included on each MSD plate (Extended Data Fig. 2). To compare measurements between plates, CSF MSD signals were normalized to the diluent-only signal of their respective plates, with a ratio >1 indicating elevated cryptic HDGFL2 signal. These normalized values were then used to calculate cryptic HDGFL2 concentration based on the plate’s standard curve. The limit of detection (LoD) for the CSF:diluent signal ratio was calculated as 1.09. When converting CSF MSD signals to cryptic HDGFL2 concentrations, ratios below the LoD were denoted as 0 ng ml^−1^ cryptic HDGFL2.

With our MSD ELISA we analyzed CSF from a longitudinal cohort of 47 *C9orf72* mutation carriers from the National Institute on Neurological Disorders and Stroke (NINDS)^21^. This cohort included a total of 89 CSF samples, with each individual contributing one, two or three time points of CSF. This cohort of *C9orf72* mutation carriers offered a unique opportunity to assay both symptomatic and presymptomatic individuals; participants were recruited due to their mutation status, but not all had phenoconverted to symptomatic disease yet, affording the ability to evaluate how early loss of TDP-43 splicing repression may occur.

The study cohort included 32 individuals with symptomatic ALS (*n* = 21), ALS–FTD (*n* = 8) or FTD (*n* = 3) and 15 presymptomatic individuals. The mean age at collection for all CSF samples was 52.4 years (s.d. 11.3, range 28.6–72.5). Presymptomatic individuals were 44.2 years old on average (s.d. 9.3, range 28.6–59.8) while symptomatic individuals were 57.4 years old on average (s.d. 9.4, range 35.7–72.5).

Of the 47 subjects, 23 (48.9%) were female with 45 of 89 (50.6%) CSF samples collected from females. Of the 45 CSF samples from females, 26 (57.8%) were collected from the presymptomatic stage of ALS while 19 (42.2%) were collected from the symptomatic stage. Of the 44 CSF samples from males, eight (18.2%) were collected from the presymptomatic stage of ALS while 17 (38.6%) were collected from ALS, six (13.6%) from FTD and 13 (29.5%) from ALS–FTD. Thus, among presymptomatic stage CSF samples, 26 of 34 (76.5%) were collected from females and, among symptomatic stage CSF samples, 19 of 55 (34.5%) were collected from females. Average MSD cryptic HDGFL2 CSF:diluent signal ratios were not significantly different between males (mean, 5.9, s.d. 14.8, range 0.73–71.20) and females (mean 1.3, s.d. 0.99, range 0.74–5.30; *P* = 0.35), although levels trended higher in males.

**Extended Data Fig. 3:**
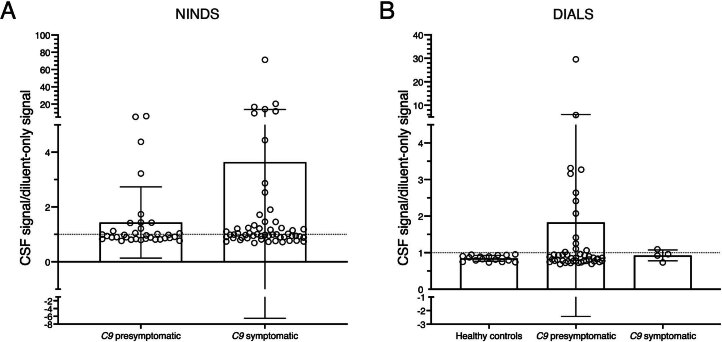
Cryptic HDGFL2 levels measured by MSD in *C9orf72* mutation carriers from NINDS and DIALS cohorts. (**A**) NINDS cohort CSF signal/diluent-only signal ratios. Of the symptomatic group (mean = 3.6, SD = 10.1, range = 0.69–71.2), 27/55 (49.1%) CSF samples had CSF/diluent signal ratios greater than 1, indicating elevated cryptic HDGFL2 signal. Of the presymptomatic group (mean = 1.4, SD = 1.3, range = 0.76–6.0), 13/34 (38.2%) CSF samples had CSF/diluent signal ratios greater than 1. Data are presented as mean +/- SD. Data points represent individual CSF samples. (**B**) DIALS cohort CSF signal/diluent-only signal ratios. Of the control group, 0/16 (0%) CSF samples had CSF/diluent ratios greater than 1. Of the symptomatic group, 1/4 (25.0%) CSF samples had CSF/diluent ratios greater than 1, and of the presymptomatic group, 13/47 (27.7%) CSF samples had CSF/diluent ratios greater than 1. The presymptomatic group had the highest average CSF/diluent signal ratio (mean = 1.8, SD = 4.2, range = 0.69–29.6) while the symptomatic group had the second highest average CSF/diluent signal ratio (mean = 0.93, SD = 0.15, range = 0.73–1.1). The control group had the lowest CSF/diluent signal ratio (mean = 0.85, SD = 0.08, range = 0.73–0.95). Data are presented as mean +/- SD. Data points represent individual CSF samples.

Of the symptomatic group, 27 of 55 (49.1%) CSF samples had CSF:diluent signal ratios >1, indicating elevated cryptic HDGFL2 signal (symptomatic group mean 3.6, s.d. 10.1, range 0.69–71.20; Extended Data Fig. 3a). Cryptic HDGFL2 was above the LoD, and its concentration could be calculated based on the lower range of the purified cryptic HDGFL2 standard curve, in 19 of 55 (34.5%) symptomatic CSF samples (symptomatic group mean 7.5 ng ml^−1^, s.d. 21.1 ng ml^−1^, range 0–97.9 ng ml^−1^). In the presymptomatic group, 13 of 34 (38.2%) CSF samples had CSF:diluent signal ratios >1 (presymptomatic group mean 1.4, s.d. 1.3, range 0.76–6.00; Extended Data Fig. 3a). Cryptic HDGFL2 was above the LoD, and its concentration could be calculated based on the standard curve, in ten of 34 (29.4%) presymptomatic CSF samples (presymptomatic group mean 2.7 ng ml^−1^, s.d. 6.8 ng ml^−1^, range 0–26.7 ng ml^−1^). Cryptic HDGFL2 CSF:diluent signal ratios were not significantly different between presymptomatic and symptomatic *C9orf72* mutation carriers (*P* = 0.49).

### Cryptic HDGFL2 is elevated in familial and sporadic ALS CSF

To confirm the novel finding that cryptic peptides reflecting TDP-43 loss of function are detectable in presymptomatic disease, we analyzed an additional cohort of largely presymptomatic *C9orf72* mutation carriers from the Dominant Inherited ALS (DIALS) Network to further characterize early loss of TDP-43 splicing repression. From this cohort we analyzed 16 CSF samples from 12 healthy controls, 47 CSF samples from 29 presymptomatic *C9orf72* mutation carriers and four CSF samples from three symptomatic converters (Extended Data Fig. 3b).

We also assayed CSF of 17 additional symptomatic *C9orf72* mutation carriers from the Northeast Amyotrophic Lateral Sclerosis (NEALS) Consortium. In addition, we analyzed CSF of 44 sporadic ALS cases from Biogen and NEALS to determine whether loss of TDP-43 splicing repression also occurs in sporadic ALS before end-stage disease. Furthermore, we analyzed 50 disease controls—a younger cohort who presented with migraine (*n* = 25) and an older cohort who presented with normal-pressure hydrocephalus (NPH; *n* = 25).

Of the 76 CSF samples from symptomatic *C9orf72* mutation carriers in all cohorts, 54 (71.1%) were from ALS, 13 (17.1%) from ALS–FTD and nine (11.8%) from FTD.

Seventeen sporadic ALS CSF samples had previously been thawed once before MSD analysis, while 27 sporadic ALS CSF samples had never been thawed. Of the 17 additional symptomatic *C9orf72* samples from NEALS, four had previously been thawed once while 13 had never been thawed. Disease control CSF samples had not previously been thawed. All CSF from healthy controls and presymptomatic and symptomatic *C9orf72* mutation carriers in the DIALS cohort had not been previously thawed before MSD analysis. All CSF from the presymptomatic and symptomatic *C9orf72* mutation carriers part of the NINDS cohort had previously been thawed once.

**Extended Data Fig. 4:**
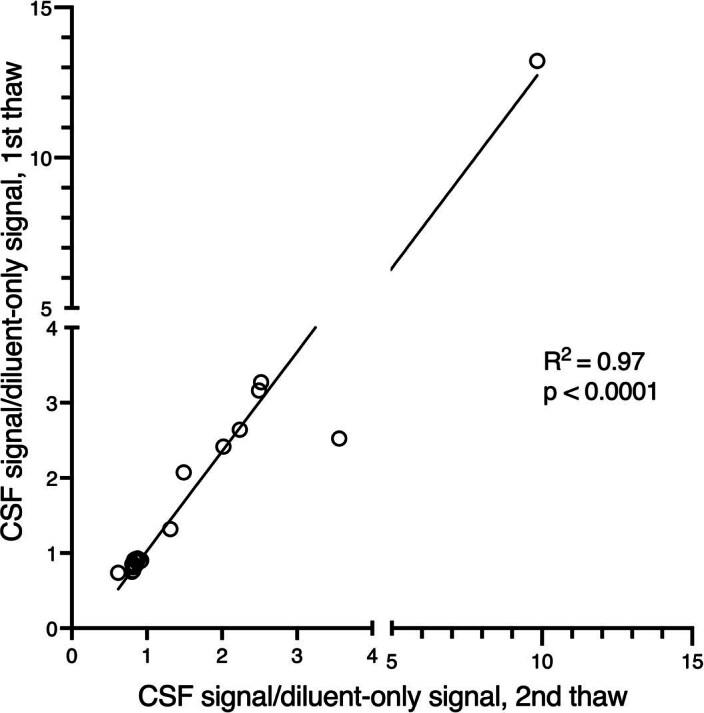
Effect of freeze-thaw on CSF cryptic HDGFL2 MSD assay. Twenty-three *C9orf72* mutation carrier CSF samples were assayed both upon first thaw and after one freeze-thaw cycle. Normalized MSD signals were slightly decreased for the previously thawed samples, but relative signal levels were preserved (simple linear regression, y = 1.324x-0.2966, R^2^ = 0.97, p < 1.0×10^−15^).

Due to differences in freeze–thaw cycles between different samples, we analyzed the effect of one freeze–thaw cycle on cryptic HDGFL2 measurements. We assayed 23 *C9orf72* mutation carrier CSF samples, following both first thaw and one freeze–thaw cycle. Normalized MSD signals were slightly decreased following one freeze–thaw cycle, but relative signal levels were preserved (simple linear regression, *y* = 1.324*x* − 0.2966, *R*^2^ = 0.97, *P* < 0.0001; Extended Data Fig. 4). We normalized CSF:diluent signal ratios of previously thawed samples according to this regression model for comparison of all CSF samples.

The mean age at sample collection was 44.0 years (s.d. 13.6, range 21.9–65.4) for healthy controls, 38.1 years (s.d. 10.4, range 17–59) for migraine controls, 71.2 years for NPH controls (s.d. 7.5, range 58–85), 43.0 years for presymptomatic *C9orf72* mutation carriers (s.d. 9.7, range 23.1–64.2), 57.6 years for symptomatic *C9orf72* mutation carriers (s.d. 9.0, range 35.7–75.4) and 59.0 years for sporadic ALS (s.d. 11.3, range 37.8–80.0) (Extended Data Table 1).

**Fig. 5:**
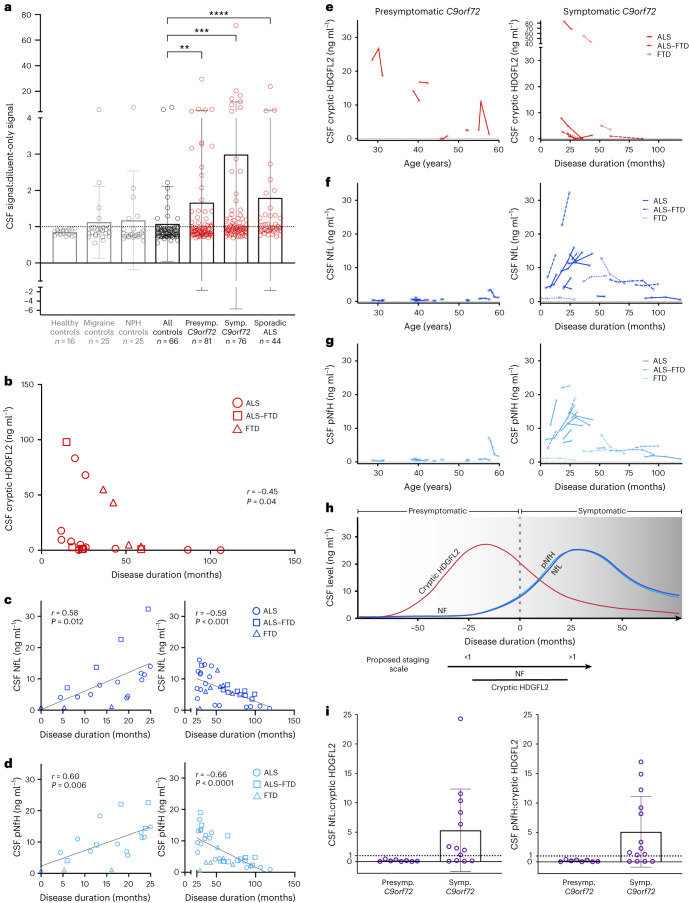
Cryptic HDGFL2 is elevated in CSF of sporadic ALS and of *C9orf72* mutation carriers, including in the presymptomatic stage. **a**, Cryptic HDGFL2 CSF:diluent MSD signal ratios measured in CSF of healthy controls (*n* = 16), migraine controls (*n* = 25), NPH controls (*n* = 25), presymptomatic (*n* = 81) and symptomatic (*n* = 76) *C9orf72* mutation carriers, and sporadic ALS (*n* = 44). Values and statistics are shown in Extended Data Table 1. Ratios >1 indicate elevated signal. Data presented as mean ± s.d. Data points represent individuals with longitudinal CSF measurements averaged as applicable. Mann–Whitney *U*-test with Holm–Bonferroni correction, *P ≤ 0.05, **P ≤ 0.01, ***P ≤ 0.001, ****P ≤ 0.0001. **b**, Cryptic HDGFL2 concentrations are negatively correlated with disease duration in NINDS symptomatic *C9orf72* mutation carriers (Spearman, two-tailed, *r* = −0.45, *P* = 0.040), suggesting that cryptic HDGFL2 levels tend to be higher earlier in disease. **c**,**d**, CSF NfL (**c**) and pNfH (**d**) concentrations are positively correlated with disease duration during the first 25 months of symptomatic disease (Pearson, two-tailed, *r* = 0.58, *P* = 0.012 (**c**); *r* = 0.60, *P* = 0.006 (**d**)) and negatively correlated with disease duration after 25 months (Pearson, two-tailed, *r* = −0.59, *P* = 0.0003 (**c**); *r* = −0.66, *P* = 1.7 × 10^−5^ (**d**). **e**–**g**, Change in CSF cryptic HDGFL2 (**e**), NfL (**f**) and pNfH (**g**) levels (ng ml^−1^) in presymptomatic *C9orf72* mutation carriers across age (left) and in symptomatic *C9orf72* mutation carriers across disease progression (right). Each line represents one individual. **h**, Proposed ALS staging model (adapted from Benatar et al.^24^) based on the dynamic of NF subunit and cryptic HDGFL2 accumulation in CSF of *C9orf72* mutation carriers. While CSF NF levels rise during the prodromal phase and continue increasing during the first few years of symptomatic disease, CSF cryptic HDGFL2 may peak before symptom onset and decrease during symptomatic disease progression. Due to these temporal differences, we propose a staging scale whereby the ratio of CSF NfL:cryptic HDGFL2 or pNfH:cryptic HDGFL2 concentrations would be <1 during the presymptomatic stage of ALS–FTD and increase to >1 during symptomatic disease. **i**, Among presymptomatic *C9orf72* mutation carriers with detectable cryptic HDGFL2 levels and available NF measurements (*n* = 8), eight of eight (100%) individuals had NfL:cryptic HDGFL2 and pNfH:cryptic HDGFL2 ratios <1. Of the symptomatic *C9orf72* mutation carriers with detectable cryptic HDGFL2 levels and available NfL (*n* = 13) or pNfH (*n* = 14) measurements, nine of 13 (69.2%) and ten of 14 (71.4%) individuals had NfL:cryptic HDGFL2 and pNfH:cryptic HDGFL2 ratios >1, respectively. Data presented as mean ± s.d.

**Extended Data Fig. 5:**
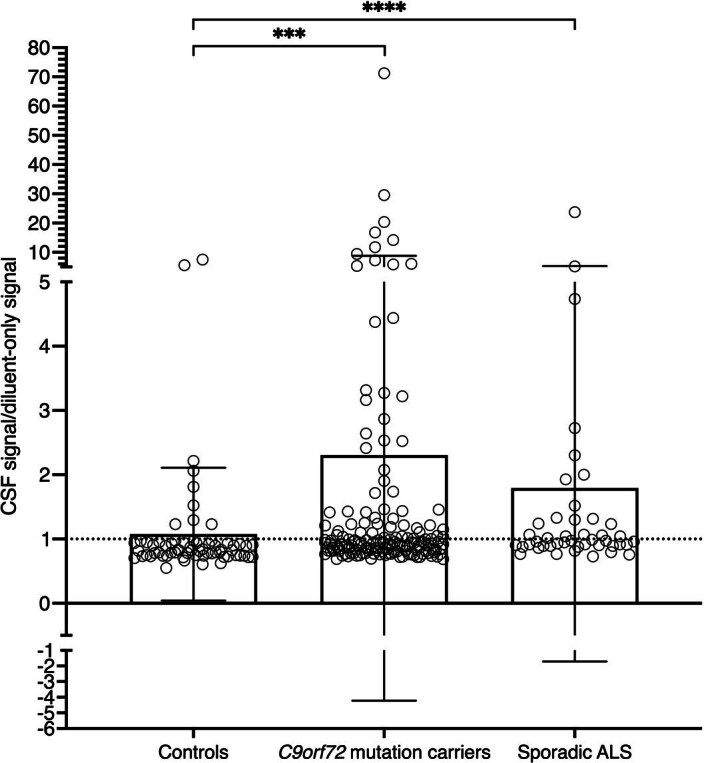
CSF cryptic HDGFL2 levels measured by MSD in all CSF samples grouped into sporadic ALS, *C9orf72* mutation carriers, and controls. The CSF/diluent ratios in sporadic ALS (n = 44 individuals, mean = 1.79, SD = 3.50; z = −4.09, p = 6x10^−5^) and *C9orf72* mutation carriers (n = 96 individuals for statistical analysis, data points show all n = 157 CSF samples, mean = 2.30, SD = 6.52, z = −3.71, p = 0.0002) were significantly higher than those in control CSF samples (n = 62 individuals for statistical analysis, data points show all n = 66 CSF samples, mean = 1.09, SD = 1.07). Where multiple CSF samples were available from one individual, CSF/diluent ratios for each sample were averaged for that individual for statistical analyses. Signal ratios were compared between disease groups and all controls with the Mann-Whitney U test (two-tailed). The Holm-Bonferroni correction was applied to all *p*-values. ***: p ≤ 0.001, ****: p ≤ 0.0001. Data are presented as mean +/- SD.

Comparing all 267 normalized CSF samples (Fig. 5a and Extended Data Table 1), symptomatic *C9orf72* mutation carriers had the highest average CSF:diluent signal ratios (mean 3.0, s.d. 8.7, range 0.69–71.20) followed by sporadic ALS (mean 1.8, s.d. 3.5, range 0.73–23.70) and presymptomatic *C9orf72* mutation carriers (mean 1.7, s.d. 3.3, range 0.69–29.60). CSF:diluent signal ratios of older, NPH controls (mean 1.2, s.d. 1.4, range 0.61–7.50) and younger, migraine controls (mean 1.1, s.d. 0.99, range 0.55–5.60) followed. Healthy controls had the lowest cryptic HDGFL2 signal ratios (mean 0.85, s.d. 0.08, range 0.73–0.95; Fig. 5a and Extended Data Table 1). When compared with all control CSF samples (mean 1.1, s.d. 1.0, range 0.55–7.50), CSF:diluent ratios in sporadic ALS (*P* < 0.0001), symptomatic *C9orf72* mutation carriers (*P* < 0.001) and presymptomatic *C9orf72* mutation carriers (*P* = 0.008) were significantly higher (Holm–Bonferroni correction; Extended Data Table 1 and Extended Data Fig. 5).

When evaluating the frequency of positive cryptic HDGFL2 signal (CSF:diluent ratio >1) in the cohort, sporadic ALS (19 of 44, 43.2%; *P* = 0.005), symptomatic *C9orf72* mutation carrier (20 of 52, 38.5%; *P* = 0.010) and presymptomatic *C9orf72* mutation carrier (16 of 44, 36.4%; *P* = 0.011) groups all had significantly higher frequencies of cryptic HDGFL2-positive individuals than the control group (nine of 62, 14.5%, Holm–Bonferroni correction; Extended Data Table 1).

### Correlations of CSF cryptic HDGFL2 with patient characteristics

We assessed the relationships of CSF cryptic HDGFL2 levels with sex, age, disease duration and revised ALS functional rating scale scores^22^.

In the overall study population there was no difference in cryptic HDGFL2 signal ratios between males and females (*P* = 0.24). In addition, there was no difference in cryptic HDGFL2 signal ratios between males and females in control (*P* = 0.91), presymptomatic *C9orf72* mutation carrier (*P* = 0.30), symptomatic *C9orf72* mutation carrier (*P* = 0.08), or sporadic ALS (*P* = 0.46) groups.

In the overall study population, cryptic HDGFL2 signal ratios and age at CSF collection were not correlated (*r* = 0.079, *P* = 0.20). In *C9orf72* mutation carriers, however, a positive correlation with age was seen (*r* = 0.18, *P* = 0.025). A correlation between cryptic HDGFL2 signal ratios and age was seen in neither sporadic ALS (*r* = 0.005, *P* = 0.97) nor controls (*r* = −0.15, *P* = 0.24).

Above-zero cryptic HDGFL2 concentrations were negatively correlated with disease duration in NINDS symptomatic *C9orf72* mutation carriers (*r* = −0.45, *P* = 0.040; Fig. 5b), suggesting that cryptic HDGFL2 levels tend to be higher earlier in disease. However, this trend was not significant when considering the smaller number of cryptic HDGFL2 concentrations above the LoD (*r* = −0.35, *P* = 0.15). Cryptic HDGFL2 concentrations above the LoD were not correlated with disease duration in sporadic ALS (*r* = −0.19, *P* = 0.60).

Elevated cryptic HDGFL2 signal ratios were not correlated with revised ALS functional rating scale scores in *C9orf72*-linked ALS or ALS–FTD (*r* = −0.31, *P* = 0.13), or in sporadic ALS (*r* = 0.36, *P* = 0.15).

### CSF cryptic HDGFL2 levels decrease in symptomatic disease

**Extended Data Fig. 6:**
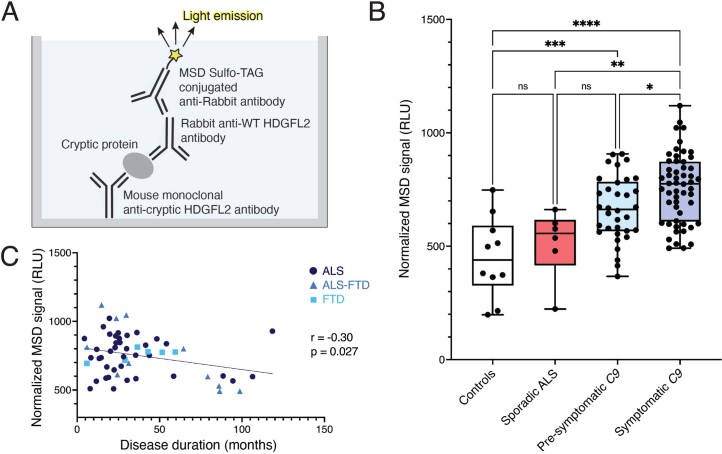
Cryptic HDGFL2 can be detected by a different MSD assay in CSF of both presymptomatic and symptomatic *C9orf72* mutation carriers. (**A**) Original sandwich ELISA using Meso Scale Discovery (MSD) system. (**B**) Elevated cryptic HDGFL2 levels are detected in presymptomatic (n = 34 CSF samples, mean = 665.4, SD = 142.5, p = 0.0009) and symptomatic (n = 54 CSF samples, mean = 758.4, SD = 152.2, p = 3.3x10^−7^) *C9orf72* mutation carriers compared to controls (n = 10 CSF samples, mean = 451.4, SD = 178.8). Elevated cryptic HDGFL2 levels are not found in the small cohort of sporadic ALS (n = 6 CSF samples, mean = 513.0, SD = 154.7, p = 0.86). The box extends from the 25^th^ to the 75^th^ percentile, with the middle line at the median. The whiskers extend from the minimum to the maximum. Analysis performed with one-way ANOVA with Tukey’s multiple comparisons test. *: p ≤ 0.05, **: p ≤ 0.01, ***: p ≤ 0.001, ****: p ≤ 0.0001. (**C**) Cryptic HDGFL2 detection by our MSD assay in CSF of *C9orf72* mutation carriers diagnosed with ALS, FTD, or ALS-FTD tends to be higher during the earlier stage of symptomatic disease. Pearson correlation (two-tailed), r = −0.30, p = 0.027.

**Extended Data Fig. 7:**
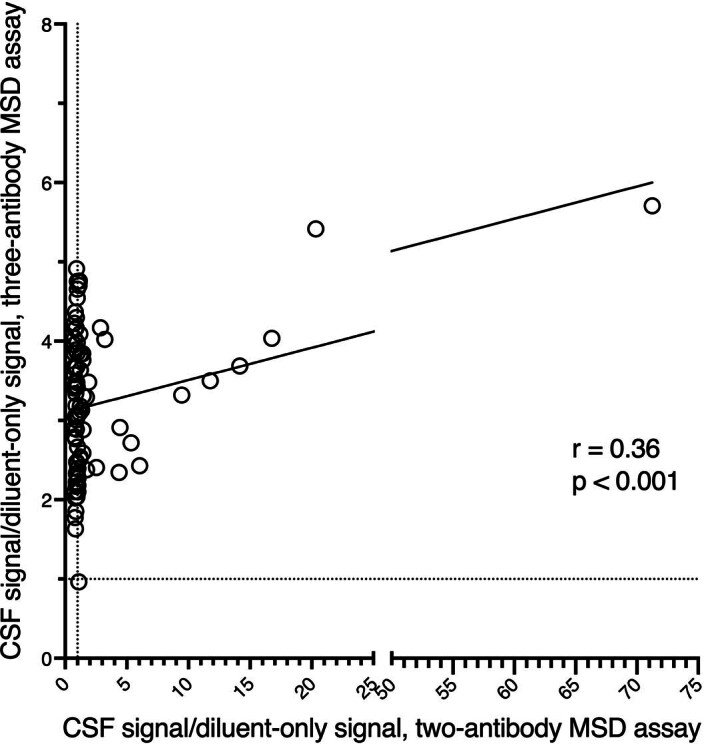
Cryptic HDGFL2 signals in *C9orf72* mutation carrier CSF samples analyzed with the current two-antibody MSD assay and a different three-antibody MSD assay are significantly correlated. Pearson correlation (two-tailed), r = 0.36, p = 0.0005. Data points show the ratios of CSF signal normalized to signal of wells containing diluent only. The current two-antibody assay demonstrates reduced background signal.

Preceding the current assay, we previously analyzed CSF of the NINDS cohort with a different version of our MSD assay. This version utilized a rabbit primary detection antibody and a sulfo-tagged, species-specific secondary detection antibody for a total of three antibodies, including the cryptic HDGFL2 capture antibody (Extended Data Fig. 6a). The data from this three-antibody assay, similarly to the two-antibody assay, showed elevated cryptic HDGFL2 signal in both presymptomatic and symptomatic *C9orf72* mutation carriers (Extended Data Fig. 6b). In addition, these signals were significantly correlated with *C9orf72* mutation carrier cryptic HDGFL2 signals from the two-antibody assay (*r* = 0.36, *P* < 0.001, Pearson), despite the two-antibody assay showing lower background (Extended Data Fig. 7). The data from the three-antibody assay showed that cryptic HDGFL2 levels were higher earlier in disease (Extended Data Fig. 6c), which was also observed with the two-antibody assay in symptomatic *C9orf72* mutation carriers as mentioned above.

Due to these data supporting an early, presymptomatic increase in cryptic HDGFL2 levels in familial ALS–FTD followed by a decline during symptomatic disease, we analyzed longitudinal CSF samples of both presymptomatic and symptomatic *C9orf72* mutation carriers from the NINDS and DIALS cohorts to elucidate the dynamics of cryptic HDGFL2 in CSF (Fig. 5e).

In eight of the nine (88.9%) symptomatic *C9orf72* mutation carriers who had detectable concentrations of cryptic HDGFL2 at least once longitudinally, cryptic HDGFL2 levels decreased with later time points of CSF collection. In one of the nine individuals, cryptic HDGFL2 levels decreased from the first to the second sample collection but increased from the second to the third (Fig. 5e).

In the six presymptomatic *C9orf72* mutation carriers who had detectable concentrations of cryptic HDGFL2 at least once longitudinally, cryptic HDGFL2 levels did not display such a unidirectional dynamic. In three of the individuals cryptic HDGFL2 decreased longitudinally while in two others cryptic HDGFL2 levels displayed a peak, increasing and subsequently decreasing. The last individual showed an increase in cryptic HDGFL2 signal from a previously undetectable signal (Fig. 5e).

Taken together, these data suggest that CSF cryptic HDGFL2 tends to decrease during symptomatic disease and may peak presymptomatically. In contrast, CSF neurofilament light chain (NfL) levels have been shown to increase in the ~12 months preceding clinically overt ALS and to continue increasing through at least the first 6 months following symptom onset^23^.

For comparison of neurofilament (NF) and cryptic HDGFL2 levels in the same cohort we analyzed CSF NfL and phosphorylated NF heavy chain (pNfH) in the NINDS cohort *C9orf72* mutation carriers. Early in symptomatic disease—within the first 25 months of disease onset—*C9orf72* mutation carriers displayed a positive correlation between disease duration and levels of both NfL (*r* = 0.58, *P* = 0.012; Fig. 5c) and pNfH (*r* = 0.60, *P* = 0.006; Fig. 5d). While increases in CSF NF levels during this initial phase are expected^23,24^, later in symptomatic disease (after 25 months) *C9orf72* mutation carriers showed a negative correlation between disease duration and levels of both NfL (*r* = −0.59, *P* < 0.001; Fig. 5c) and pNfH (*r* = −0.66, *P* < 0.0001; Fig. 5d). To better understand these relationships we also analyzed longitudinal dynamics of CSF NfL and pNfH. For symptomatic *C9orf72* mutation carriers within 50 months of disease onset, ten of 13 (76.9%) and 11 of 14 (78.6%) individuals showed overall longitudinal increases in NfL (Fig. 5f) and pNfH (Fig. 5g), respectively. For symptomatic *C9orf72* mutation carriers >50 months from disease onset, four of five (80%) and three of five (60%) individuals displayed overall longitudinal decreases in levels of NfL (Fig. 5f) and pNfH (Fig. 5g), respectively. In presymptomatic *C9orf72* mutation carriers, NfL and pNfH levels remained low except for those in the oldest individual (Fig. 5f,g). These data suggest that CSF NfL and pNfH levels increase during the first few years following clinical diagnosis and may later decrease. In contrast, CSF cryptic HDGFL2 levels tend to decrease during the first few years of symptomatic disease, potentially having peaked during the presymptomatic stage. CSF cryptic HDGFL2 and NF levels thus tend to follow similar trajectories, with the cryptic HDGFL2 peak occurring earlier than that of NFs (Fig. 5h).

Due to the temporal differences between increases in CSF levels of cryptic HDGFL2 and NF subunits, we propose a staging scale whereby the ratio of CSF NfL:cryptic HDGFL2 or pNfH:cryptic HDGFL2 concentrations (ng ml^−1^) would be <1 during the presymptomatic stage of ALS–FTD and increase to >1 during symptomatic disease (Fig. 5h). Of the presymptomatic *C9orf72* mutation carriers with detectable cryptic HDGFL2 levels and available NF measurements, eight of eight (100%) individuals had NfL:cryptic HDGFL2 and pNfH:cryptic HDGFL2 ratios <1 (Fig. 5I). Of the symptomatic *C9orf72* mutation carriers with detectable cryptic HDGFL2 levels and available NF measurements, nine of 13 (69.2%) and ten of 14 (71.4%) individuals had NfL:cryptic HDGFL2 and pNfH:cryptic HDGFL2 ratios >1, respectively (Fig. 5i).

### Cryptic HDGFL2 is detectable in presymptomatic *C9orf72* blood

**Fig. 6:**
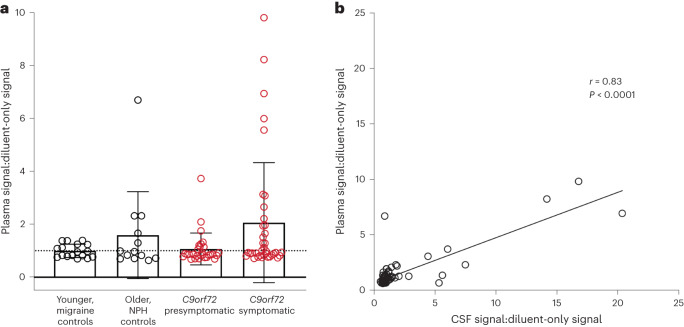
Cryptic HDGFL2 can be detected by MSD assay in plasma of both presymptomatic and symptomatic *C9orf72* mutation carriers. **a**, In the symptomatic group (mean 2.1, s.d. 2.3, range 0.71–9.80), 17 of 37 (45.9%) plasma samples had cryptic HDGFL2 plasma:diluent MSD signal ratios >1. In the presymptomatic group (mean 1.1, s.d. 0.60, range 0.68–3.70), nine of 29 (31.0%) plasma samples had plasma:diluent signal ratios >1. The difference between symptomatic and presymptomatic *C9orf72* mutation carriers was not significant (*z* = −0.82, *P* = 0.42, Mann–Whitney *U*-test). Cryptic HDGFL2 levels were also measured in older controls with NPH (*n* = 13, mean 1.6, s.d. 1.6, range 0.64–6.70) and in younger controls with migraine (*n* = 17, mean 1.0, s.d. 0.25, range 0.69–1.40). Data presented as mean ± s.d. Data points represent individual plasma samples. **b**, Plasma:diluent signal ratios were significantly correlated with CSF:diluent signal ratios from matching CSF samples (*r* = 0.83, *P* < 1.0 × 10^−15^, Pearson, two-tailed).

**Extended Data Fig. 8:**
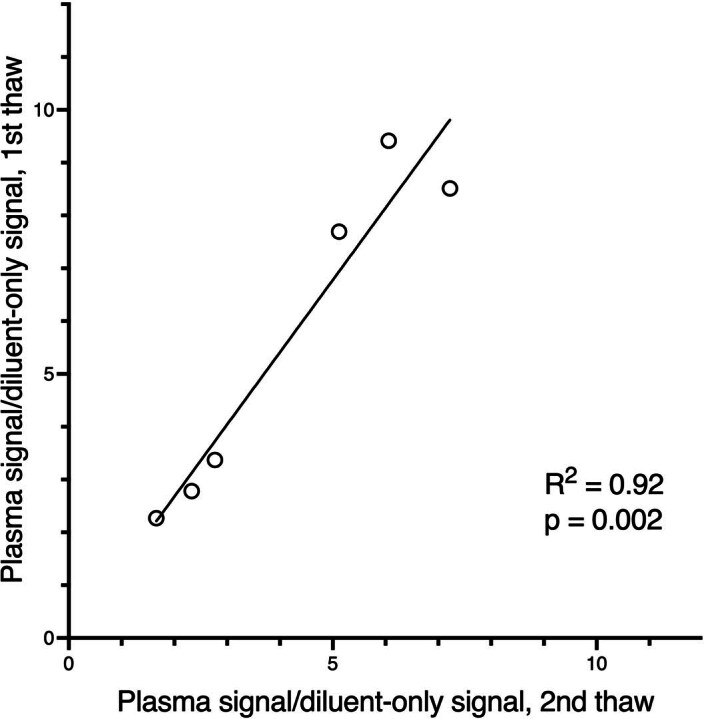
Effect of freeze-thaw on plasma cryptic HDGFL2 MSD assay. Six *C9orf72* mutation carrier plasma samples were assayed both upon first thaw and after one freeze-thaw cycle. Normalized MSD signals were slightly decreased for the previously thawed samples, but relative signal levels were preserved (simple linear regression, y = 1. 366x-0.05577, R^2^ = 0.92, p = 0.002).

In addition to CSF, we used our novel MSD assay to measure cryptic HDGFL2 levels in 66 plasma samples from 43 *C9orf72* mutation carriers in the NINDS cohort (Fig. 6a). Six samples had previously been thawed once while the others had never been thawed. Normalized MSD signals were slightly decreased following one freeze–thaw cycle, but relative signal levels were preserved (simple linear regression, *y* = 1.366x − 0.05577, *R*^2^ = 0.92, *P* = 0.002; Extended Data Fig. 8). We normalized plasma:diluent signal ratios of the six previously thawed samples according to this regression model to compare all plasma samples.

The study cohort included 28 individuals with symptomatic ALS (*n* = 22), ALS–FTD (*n* = 4) or FTD (*n* = 2) and 15 presymptomatic individuals. The mean age from all *C9orf72* mutation carrier plasma collections was 52.0 years (s.d. 10.7, range 28.6–72.5). Of the 43 individuals 23 (53.5%) were female, with 37 of 66 (56.1%) plasma samples collected from females. Of the 37 plasma samples from females, 22 (59.5%) were collected from the presymptomatic stage of ALS while 15 (40.5%) were collected from the symptomatic stage. Of the 29 plasma samples from males, seven (24.1%) were collected from the presymptomatic stage while 14 (48.3%) were collected from ALS, two (6.9%) from FTD and six (20.7%) from ALS–FTD. There was no significant difference between ratios of plasma signal:diluent signal in males (mean 2.3, s.d. 2.5, range 0.68–9.80) and females (mean 1.1, s.d. 0.57, range 0.69–3.70; *P* = 0.43).

In the symptomatic group, 17 of 37 (45.9%) plasma samples had plasma:diluent signal ratios >1. In the presymptomatic group, nine of 29 (31.0%) plasma samples had plasma:diluent signal ratios >1. Cryptic HDGFL2 plasma:diluent signal ratios were higher in symptomatic (mean 2.1, s.d. 2.3, range 0.71–9.80) compared with presymptomatic *C9orf72* mutation carriers (mean 1.1, s.d. 0.60, range 0.68–3.70), but the difference was not significant (*P* = 0.42).

Thirty control plasma samples were also analyzed (Fig. 6a). This control group comprised 13 samples from the older, disease control cohort of individuals with NPH and 17 from the younger, disease control cohort of individuals with migraine. The mean age at sample collection for the older controls was 71.5 years (s.d. 7.4, range 58–84) and that for younger controls was 38.4 years (s.d. 9.3, range 17–51). Cryptic HDGFL2 levels, measured as plasma:diluent signal ratios, in older controls (mean 1.6, s.d. 1.6, range 0.64–6.70) and younger controls (mean 1.0, s.d. 0.25, range 0.69–1.40) were not significantly different (*P* = 0.70).

The LoD for plasma:diluent signal ratio was calculated as 1.11. When converting plasma MSD signals to cryptic HDGFL2 concentrations, ratios below the LoD were denoted as 0 ng ml^−1^ cryptic HDGFL2. Symptomatic *C9orf72* mutation carriers had the highest concentrations of cryptic HDGFL2 (mean 6.4 ng ml^−1^, s.d. 11.4, range 0–42.7 ng ml^−1^), and individuals with NPH had the next-highest concentrations (mean 4.8 ng ml^−1^, s.d. 9.5, range 0–34.1 ng ml^−1^) followed by presymptomatic *C9orf72* mutation carriers (mean 1.3 ng ml^−1^, s.d. 3.5, range 0–17.1 ng ml^−1^). The migraine cohort had the lowest concentrations (mean 1.1 ng ml^−1^, s.d. 1.6, range 0–3.7 ng ml^−1^). These differences were not significant, however.

**Extended Data Fig. 9:**
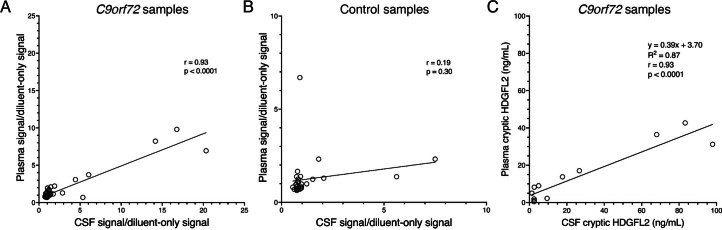
CSF and plasma cryptic HDGFL2 levels are highly correlated in *C9orf72* mutation carriers. (**A**) Pearson correlation (two-tailed) of plasma and CSF cryptic HDGFL2 signal ratios in *C9orf72* mutation carriers (n = 57 matching plasma/CSF samples), r = 0.93, p < 1.0x10^−15^. One CSF ratio outlier removed. (**B**) Pearson correlation (two-tailed) of plasma and CSF cryptic HDGFL2 signal ratios in migraine and NPH disease controls (n = 30 individuals/matching plasma and CSF samples), r = 0.19, p = 0.30. CSF and plasma signals are not correlated in controls. (**C**) Non-zero CSF and plasma cryptic HDGFL2 concentrations are correlated in *C9orf72* mutation carriers (n = 12 matching plasma/CSF samples). Pearson correlation (two-tailed), r = 0.93, p = 8.3x10^−6^. The equation for the line of best fit was y = 0.39x + 3.70 (linear regression, R^2^ = 0.87, p = 8.3x10^−6^). Higher concentrations of cryptic HDGFL2 can be found in CSF than in plasma.

Fifty-eight *C9orf72* mutation carrier plasma samples had previously assayed matching CSF samples from the NINDS cohort, and the 30 control plasma samples also had previously assayed matching CSF samples. Plasma:diluent signal ratios were highly correlated with CSF:diluent signal ratios (*r* = 0.83, *P* < 0.0001, Pearson; Fig. 6b). When analyzing the two groups separately, plasma and CSF cryptic HDGFL2 levels were highly correlated in *C9orf72* mutation carriers (*r* = 0.93, *P* < 0.0001, Pearson; Extended Data Fig. 9a) but not in controls (*r* = 0.19, *P* = 0.30, Pearson; Extended Data Fig. 9b). When comparing nonzero concentrations (ng ml^−1^) of cryptic HDGFL2, plasma and CSF measurements were again highly correlated in *C9orf72* mutation carriers (*r* = 0.93, *P* < 0.0001, Pearson; Extended Data Fig. 9c). These results indicate that CSF and plasma levels of cryptic HDGFL2 have a strong linear relationship in *C9orf72*-linked disease. Higher concentrations of cryptic HDGFL2 can be found in CSF than in plasma, however (Extended Data Fig. 9c).

## Discussion

Previously we proposed that loss of TDP-43 splicing repression of cryptic exons underlies the disease pathogenesis of ALS–FTD^7^. This view is further supported by the identification of key cryptic exon targets of TDP-43, such as *UNC13A* and *STMN2* (refs. ^9,11–14,25–27^). However, because no method currently exists for the detection of TDP-43 dysfunction in living individuals, evidence that such loss of TDP-43 function occurs during early-stage disease rather than being an end-stage phenomenon remains elusive.

Our findings demonstrating the presence of cryptic HDGFL2 in both CSF and blood of individuals with ALS–FTD now provide direct evidence that loss of TDP-43 splicing repression occurs during early-stage disease, including the presymptomatic phase. These findings align with a case in which a patient underwent surgical resection of her temporal lobe for treatment of epilepsy 5 years before developing FTD symptoms. Her resected brain tissue was shown to have lost nuclear TDP-43 in some neurons but did not contain any cytoplasmic inclusions, supporting the notion that loss of TDP-43 splicing repression represents an early event that drives neuron loss^28^. Furthermore, another group was also able to detect the presence of cryptic HDGFL2 in CSF of presymptomatic *C9orf72* mutation carriers, instead using a targeted mass spectrometry approach^29^. Our detection of TDP-43-dependent cryptic peptides in CSF and blood of presymptomatic *C9orf72* mutation carriers also aligns with a common theme of neurodegenerative diseases—that pathogenic mechanisms often begin years before the emergence of clinically relevant symptoms.

Our analysis of cryptic peptide accumulation in longitudinal CSF of numerous *C9orf72*-linked individuals allows us not only to establish loss of TDP-43 splicing repression occurring at a presymptomatic stage, but also to discern the dynamic nature of TDP-43-dependent cryptic proteins in CSF. We propose that cryptic HDGFL2 levels may peak presymptomatically in some individuals and decrease as disease progresses, and thus measurement of CSF cryptic HDGFL2 may provide the ability to predict phenoconversion in familial ALS–FTD, particularly when evaluated in combination with CSF NF levels.

Our data show that, in *C9orf72* mutation carriers, CSF levels of cryptic HDGFL2 and NF subunits tend to follow similar trajectories, with the increase in cryptic HDGFL2 occurring before those of NfL and pNfH. Previous studies suggest that NfL and pNfH levels may begin to rise shortly before symptom onset in ALS and later plateau during symptomatic disease^23,24,30,31^. However, these studies are limited to the first few years of symptomatic disease, and NF data beyond 50 months of symptomatic disease remain sparse. The unique longitudinal cohort of *C9orf72* mutation carriers studied here offers the opportunity to analyze NF levels beyond 50 months following disease onset in many individuals. These data suggest that CSF NfL and pNfH levels may decrease in late-stage disease. The negative correlations shown here between disease duration and NF levels following 25 months of symptomatic disease may, however, be impacted by lower survival rates in individuals with high baseline NF levels^30–32^. Further longitudinal studies of NF levels extending beyond the first few years of symptomatic disease will be important for clarification of these dynamics.

Nevertheless, the relative dynamics of cryptic HDGFL2 and NF levels shown here—where cryptic HDGFL2 levels peak before those of NF and tend to decrease during the earliest phase of symptomatic disease as NF levels are rising—suggest that these biomarkers used in conjunction could provide greater insight into phenoconversion timing. The study of longitudinal cryptic HDGFL2 trajectories relative to NfL and pNfH trajectories in additional familial ALS–FTD cohorts, as well as in sporadic ALS, will be informative. Overall, more extensive longitudinal studies are warranted to clarify the proposed biomarker dynamics (Fig. 5h) in individuals throughout their lifetimes, particularly comparing familial versus sporadic disease.

Our findings of a negative correlation of cryptic HDGFL2 levels with disease duration yet a positive correlation with age in *C9orf72* mutation carriers suggest that, while there is a tendency for cryptic HDGFL2 levels to decrease during symptomatic disease progression, there is also a population-level increased vulnerability to TDP-43 dysfunction with aging. The underlying mechanism for the decrease in CSF cryptic HDGFL2 levels during symptomatic disease progression is currently unclear. However, these changes could be related, for example, to a flurry of cell death before symptom onset, alterations in waste removal dynamics in the CNS or shifts in HDGFL2 expression.

Although further studies are needed, cryptic HDGFL2 shows promise as a new biomarker for early-stage ALS–FTD. NF markers reflect broad neurodegeneration and are elevated in many diseases, limiting their diagnostic utility, and they do not reflect TDP-43 dysfunction, limiting their ability to measure target engagement in clinical trials. Other *C9orf72*-related biomarkers have also been studied, such as dipeptide repeats^33,34^, but the functional relevance of these proteins remains unclear. Thus, cryptic peptide biomarkers would transform our current biomarkers of ALS–FTD through their early elevation, their specificity for TDP-43-related disease and their reflection of TDP-43 dysfunction.

While further study of individuals with sporadic ALS is warranted, our findings suggest that cryptic exon-encoded peptides could serve as biomarkers to facilitate earlier diagnosis of ALS, which is currently delayed by the need to rule out mimic conditions^35^. The strong correlation of cryptic HDGFL2 levels in plasma with those in CSF supports the potential utility of blood-based cryptic protein biomarkers. Due to the relative ease of collecting blood samples compared with CSF, these findings expand the value of cryptic protein biomarkers in the early stage—and even presymptomatic or prodromal phases—of disease. Such early-stage biomarkers that can identify ALS–FTD-related pathogenic mechanisms are currently critically lacking. Earlier diagnosis would permit prompt therapeutic intervention with a greater chance of success.

In addition to establishing a potential new diagnostic biomarker, our finding that loss of TDP-43 splicing repression occurs during the presymptomatic phase of disease provides a strong rationale for the development of therapeutic strategies to complement TDP-43 splicing repression deficits for ALS—for example, an AAV9 gene therapeutic strategy that expresses a splicing repressor, termed CTR^36^. Because detection of cryptic exon-encoded peptides in patient biofluids reflects loss of TDP-43 function, evaluating the dynamics of cryptic peptide biomarkers could provide a way of measuring target engagement for new therapeutics aimed at restoration of TDP-43 function. Although cryptic HDGFL2 levels decrease later in symptomatic disease, early—even presymptomatic—disease offers the best treatment window. Thus, measurement of cryptic HDGFL2 levels could be useful for evaluation of target engagement during these optimal therapeutic windows. Many clinical trials for ALS treatments currently suffer from an inability to measure therapeutic efficacy at a mechanistic level^37^, so TDP-43-dependent cryptic peptide biomarkers could be used in the future to better interpret results of clinical trials and improve drug design.

Furthermore, involvement of TDP-43 dysfunction early in *C9orf72* ALS–FTD suggests that the current standard of detection of TDP-43 pathology in postmortem tissues may miss cases of TDP-43 misregulation. TDP-43 pathology is identified by cytoplasmic inclusions reactive to anti-TDP-43 or anti-phosphorylated TDP-43 antibodies. However, a lack of overt cytoplasmic staining of TDP-43 aggregates does not rule out loss of TDP-43 splicing repression. Inclusion of cryptic exons early in disease indicates that identification of TDP-43 nuclear depletion or TDP-43-related cryptic proteins in histological sections may provide a more sensitive approach to classification of TDP-43 pathology.

In the future, development of a highly sensitive, multiplexed MSD assay to measure cryptic HDGFL2 and other disease-relevant markers simultaneously, such as pNfH, NfL, tau, amyloid-β and α-synuclein, would provide additional insight into disease staging for ALS and other neurodegenerative diseases exhibiting TDP-43 pathology. This multiplexed system will also allow us to analyze several cryptic neoepitopes at once. We envision the ability to detect a set of TDP-43-associated cryptic exon targets that may display different dynamics throughout disease progression. Analysis of the changes of these targets throughout disease could provide detailed information on disease staging, progression and potentially prognosis that is not currently available for ALS–FTD.

Analysis of larger, longitudinal cohorts with our plasma-based assay will be warranted. Although CSF and plasma cryptic HDGFL2 levels are highly correlated in *C9orf72* mutation carriers, some individuals displayed positive signal in either CSF or plasma but not in both (Extended Data Fig. 9). Comparison of cryptic HDGFL2 concentrations in CSF and plasma suggests that greater concentrations of cryptic HDGFL2 can be found in CSF, but analysis of greater numbers of matching CSF and plasma samples will help elucidate how cryptic HDGFL2 dynamics may vary in CSF and blood. Background signal levels may vary more between plasma samples than CSF samples due to matrix effects, and thus a plasma:diluent signal ratio >1 may provide a more accurate threshold for cryptic HDGFL2 positivity. Further testing will be required to clarify the contributions of assay sensitivity, matrix effects and cryptic HDGFL2 dynamics between CSF and blood measurements.

Due to the prevalence of TDP-43 dysfunction in both aging^38^ and a number of different diseases^39^, many samples of the ‘control’ groups analyzed here, particularly the older NPH control group, may not be true controls. Using a forensic autopsy cohort, a recent study showed that TDP-43 nuclear clearance can be found in the brains of a few individuals in their 50s^40^. Our finding of detectable cryptic HDGFL2 levels in some controls is thus unsurprising. Analysis of antemortem biofluids paired with postmortem pathological staining from the same individuals in both ALS–FTD and controls will be important for further validation of this assay and determination of its sensitivity and specificity.

In the future other ELISA platforms, such as NULISA^41^, should be explored to further increase the sensitivity of this assay. Although assay sensitivity could probably be improved, our findings here provide evidence that measurement of TDP-43-dependent cryptic neoepitopes in CSF and blood is possible. Future studies exploring larger cohorts in a longitudinal manner will be useful to further establish the utility of these biomarkers.

The impact of this work also extends beyond the ALS–FTD spectrum. Because many cases of Alzheimer’s disease (AD) also possess TDP-43 pathology^42–46^, cryptic peptide biomarkers would be helpful in distinguishing ‘pure AD’ exhibiting only canonical pathologies of β-amyloidosis and tauopathy from mixed (or multiple)-etiology dementia, which probably warrant different treatment strategies. Cryptic peptide biomarkers could be used in the future to stratify mixed-etiology dementia and pure AD for clinical trials, giving therapeutics the best chance of success. They could also be used retrospectively to assess whether subgroups of previous clinical trials display different therapeutic efficacy than when analyzed as a single disease group. Several other neurological conditions involve TDP-43 proteinopathy, including LATE^47^, multiple sclerosis^48^ and chronic traumatic encephalopathy^49^; therefore, the impact of these biomarkers could be far reaching.

## Methods

### Analysis of RNA sequencing data

FASTQ files were downloaded from the NCBI’s Sequence Read Archive and aligned to the GRCh38 human genome assembly using STAR (v.2.7.10a) with default parameters. Python was used to set up alignment scripts. Megadepth was used to convert the output BAM files to BigWig files, and the data were then displayed on the University of California, Santa Cruz (UCSC) Genome Browser (http://genome.ucsc.edu/) for visualization of cryptic exons^50–55^.

The data table containing normalized area under the curve (NAUC) information was downloaded from ASCOT^19^. Data were subset to the genes of interest and a heatmap was generated using the ggplot2 package in R.

### Protein structure generation and comparison

Protein sequences were identified by translation of mRNA sequences for *ACTL6B*, *AGRN*, *EPB41L4A*, *HDGFL2* and *SLC24A3* with and without the cryptic exon. WT mRNA sequences are available on GENCODE v.43 (ref. ^56^): *ACTL6B* (ENST00000160382.10), *AGRN* (ENST00000379370.7), *EPB41L4A* (ENST00000261486.6), *HDGFL2* (ENST00000616600.5) and *SLC24A3* (ENST00000328041.11).

The predicted WT protein structures for ACTL6B, EPB41L4A, HDGFL2 and SLC24A3 were downloaded from the AlphaFold Protein Structure Database^20,57^. The remaining protein structures were generated using the AlphaFold Monomer v.2.0 pipeline (v.2.2.0) on the Rockfish cluster at Johns Hopkins University. Anaconda was used to set up the AlphaFold environment. Due to the size of the AGRN protein, a truncated version of the WT protein was generated corresponding to amino acids 360–1,097 in the WT protein. Cryptic ACTL6B, EPB41L4A, HDGFL2 and SLC24A3 proteins were generated in their entirety. A truncated version of AGRN was generated that included the cryptic epitopes along with flanking amino acids corresponding to amino acids 360–1,097 in the WT protein. Pymol was used to visualize the structures.

### Generation of monoclonal cryptic antibodies

Hybridoma lines were generated by CDI Laboratories, Inc. Mice were immunized with the cryptic peptide of interest, and hybridomas were produced from these mice. IgG-positive hybridomas were identified by ELISA. Hybridoma lines were identified that produced antibodies recognizing their cognate antigen as the top target on the HuProt human protein microarray, which contains >19,500 affinity-purified recombinant human proteins^58^. Promising hybridoma lines were further screened by protein blot. Cryptic antibody no. 1-69 was purified from its hybridoma line by the Wong laboratory, and bulk purification of TC1HDG antibody from the 1-69 hybridoma line was performed by Bio X Cell following established protocols.

### Generation of goat antibody against WT HDGFL2

Keyhole limpet hemocyanin-conjugated peptide antigens (HDGFL2 amino acid sequence: AEVYTRLKSRVLGPKIEAVQC) were synthesized by the Johns Hopkins Synthesis and Sequencing Core. Polyclonal goat antibodies (gTEA1.2) were generated by Rockland Immunochemicals using established protocols.

### Generation of siTDP and control HeLa lysates

HeLa cells were seeded in six-well plates and then either transfected with a custom-order MISSION endoribonuclease-prepared siRNA targeting Human tardbp (catalog no. EHU109221) (siTDP) or not transfected (control). Transfection was performed following the Lipofectamine 3000 Reagent protocol (ThermoFisher). Per well, 2,500 ng of siRNA was combined with 125 μl of OptiMem and the mixture added to 125 μl of OptiMem with 5 μl of Lipofectamine 3000 reagent. After 15 min, 250 μl of this mixture was added slowly to each desired well. After 48 h medium was aspirated and cells washed with 1× PBS. Additional PBS was added to wells, and cells were scraped into 1.5-ml Eppendorf tubes. Cells were pelleted at 500*g* for 5 min and the supernatant removed. The pellet was resuspended in either RIPA Lysis and Extraction Buffer or M-PER Mammalian Protein Extraction Reagent with 1× protease inhibitor cocktail and kept on ice for 5 min. Cells were then centrifuged at 8,000*g* for 10 min and the supernatant collected and kept at −80 °C.

### Generation of WT and cryptic *HDGFL2* expression vectors

The WT *HDGFL2* mRNA sequence (ENST00000616600.5) was identified using UCSC Genome Browser. RNA sequencing visualization of TDP-43-knockdown motor neurons^11^ on UCSC Genome Browser was used to extract the cryptic exon sequence.

For both expression vectors, codons corresponding to a glycine serine linker and 6-His Tag (GGGSHHHHHH) were added to the 3’ end of the sequence directly before the stop codon. A Kozak sequence was added to the 5’ end of the sequence. For the WT sequence, a start codon with a twin-strep sequence (MSAWSHPQFEKGGGSGGGSGGSAWSHPQFEK) and a TEV protease cleavage site (ENLYFQG) was added to the 5’ end of the sequence after the Kozak sequence but before the original start codon.

Codons were optimized by using IDT Codon Optimizer to identify and modify codons that led to nucleic acid repeats of four or more while retaining the amino acid translation. Restriction sites corresponding to NsiI, XmnI, BclI, BstXI, NheI, BspEI, XhoI, XbaI, PspOMI, BglII, NotI and BamHI were removed from the sequence. The resulting sequence was synthesized into the pTwist CMV Puro expression vector by Twist Bioscience.

### WT and cryptic HDGFL2 protein expression/purification

tsA201 cells derived from HEK293 were purchased from Millipore Sigma (catalog no. 96121229-1VL). They were tested for mycoplasma at the European Collection of Authenticated Cell Cultures, and the identities of tsA201 and HEK293 were confirmed by short tandem repeat (STR) profiling. Cells were were adapted to growth in suspension using Gibco FreeStyle 293 Expression Medium plus 2% fetal bovine serum, 50 U ml^−1^ penicillin/streptomycin and 2 mM glutamine in shake flasks. The low passage (P2 or P3) of the suspension culture was collected and cell stock was made with the addition of 7.5% DMSO followed by storage in liquid nitrogen. For each expression, one frozen vial with 1 × 10^7^ tsA201 cells was inoculated to 40 ml of the above-mentioned medium in a 250-ml flat-bottom cell culture shaker flask. Cell density was measured using NucleoCounter NC-100 following growth for 3 days in an Infors Multitron incubator at 130 r.p.m. under 8% CO_2_ and 75% humidity. Cells were then transferred to a large flask (2.8- or 5-l Optimum Growth flask) with the addition of the above-mentioned medium to reach a final cell density of 1.3 × 10^5^ cells ml^−1^. The total volume of culture was no more than 1.0 l for a 2.8-l flask and no more than 2.1 l for a 5-l flask. Once cells had reached a density of 2 × 10^6^ ml^−1^—normally after 3 days of growth—transfection was begun. For a 1-l culture, 1 mg of pTwist CMV Puro-HDGFL2 plasmid (filtered with a 0.22-µm filter) was added to 50 ml of prewarmed Hybridoma-SFM (ThermoFisher, no. 12045076). Four milliliters of PEI MAX (Transfection Grade Linear Polyethylenimine Hydrochloride, MW 40,000; Polysciences, no. 24765-1) at 1 mg ml^−1^ was added followed by gentle mixing to create a homogenous solution. Following incubation at room temperature for 12–15 min, the mixture was added to the culture and the flask was replaced in the incubator. Four hours following transfection, sodium butyrate was added to the flask to a final concentration of 10 mM. After 2 days the culture was centrifuged at 466*g* for 15 min using a Sorvall RC3B Plus centrifuge. Cell pellets were washed once with 1× PBS and stored at −80 °C for future purification.

Frozen cell pellets (5 g) were suspended in 50 ml of 100 mM Tris pH 8.0, 150 mM NaCl and 1 mM EDTA buffer, which also contained a cocktail of protease inhibitors (cOmplete, EDTA free, Roche). The cells were then lysed using a microfluidizer at 15,000 pounds per square inch at 4 °C. The cell lysate was centrifuged (28,145*g* for 35 min) and the supernatant filtered through a 0.22-μm filter before being loaded onto a 5-ml StrepTrap HP column (Cytiva). An AKTA fast protein liquid chromatograph with Unicorn software (GE Healthcare) was used to load the supernatant and wash the column with 10 CV of buffer at 2 ml min^−1^. The protein was then eluted with the above-mentioned buffer with 2.5 mM desthiobiotin over 10 CV. The HDGFL2-containing fractions were pooled (~8 ml) and protein concentration was measured using a NanoDrop Microvolume Spectrophotometer (Thermo Fisher Scientific). HDGFL2 was then cleaved by protease TEV to remove the N-terminal twin-strep tag. Enzymatic cleavage was completed with 1 mg of TEV per 50 mg of HDGFL2 with the addition of 1 mM DTT incubated at 4 °C overnight with rocking. The digested mixture was then loaded onto a pre-equilibrated MonoQ 5/50 GL column (Cytiva) to remove the cleaved twin-strep tag, remaining uncleaved protein and additional impurities at a flow rate of 1 ml min^−1^. The running buffer was 50 mM Tris pH 8.0 and the gradient was 5 mM min^−1^ for NaCl from 0 to 500 mM. HDGFL2-containing fractions around 300 mM NaCl were immediately collected and analyzed using SDS–polyacrylamide gel electrophoresis.

### Analysis with reverse transcription PCR

RNA was extracted from HeLa samples using TRIzol (Life Tech., no. 15596-026) and RNeasy Mini Kits (Qiagen, no. 74104). Complementary DNA was derived from total RNA using the ProtoScript II First Strand cDNA Synthesis Kit (NEB, no. E6560S). Numerous primers were designed against cryptic exon targets and screened to identify primer pairs that minimized background bands. The following primer pair was used here: HDG F primer 5’-AAGACGCCTGCGCTAAAGAT-3’ and HDG R primer 5’-GCTTCCCTCCCTTCTGATGC-3’, with an expected band weight of 269 base pairs (bp).

### Protein blot and IP–protein blot analysis

Immunoprecipitation was performed by overnight incubation of HeLa lysates with the novel no. 1-69 antibody against cryptic HDGFL2 at 4 °C, with rotation. A 50% slurry of Protein G agarose beads (Cell Signaling, no. 37478) in RIPA lysis buffer was added, and this mixture was incubated with rotation at 4 °C for 1–3 h. Bead complexes were denatured in NuPAGE LDS Sample Buffer at 70 °C for 10 min and microcentrifuged at 14,000*g* for 1 min. Samples were then analyzed by SDS–polyacrylamide gel electrophoresis and protein blot.

Protein blot analysis was performed following electrophoresis using NuPAGE 4–12% Bis-Tris polyacrylamide gels for all protein blots, except for that shown in Fig. 4c, where electrophoresis was performed on Novex 6% Tris-Glycine polyacrylamide gels. Proteins were transferred onto polyvinylidene difluoride membranes using either a mini gel tank wet transfer method or the iBlot Dry Blotting System from Invitrogen. Following blocking of the membrane in 5% milk in Tris-buffered saline with 0.1% Tween, primary antibodies were incubated overnight at 4 °C with rocking. Varying concentrations were used for different primary antibodies: TDP-43, 1:4,000; GAPDH, 1:1,000; TC1HDG, 1 μg ml^−1^; HPA044208 WT HDGFL2, 1:1,000; gTEA1.2, 1 μg ml^−1^. Secondary antibodies (goat anti-mouse IgG horseradish peroxidase (HRP) 32230, goat anti-rabbit IgG HRP BP-9100-50 and bovine anti-goat IgG HRP 805-035-180) were used at either 1:10,000 or 1:20,000.

### Immunofluorescent staining of brain tissues

Postmortem human paraffin-embedded brain tissue sections from motor cortex and hippocampus were obtained from the Johns Hopkins Brain Resource Center. All participants agreed to autopsy before their death, and their next of kin consented to the autopsy procedure at the time of death. All brains were examined in the Division of Neuropathology at Johns Hopkins under a protocol approved by the JHU Institutional Review Board.

Immunofluorescent staining was performed for colabeling of cryptic HDGFL2 with total TDP-43 and phosphorylated TDP-43 on 10-μm sections from formalin-fixed tissue blocks. Each section was deparaffinized with xylene and rehydrated with graded alcohols and water. Antigen retrieval was performed using HistoVT One (Nacalai Tesque) at 95 °C for 30 min. All sections were blocked with 3% normal goat serum in PBS solution with 0.2% Triton X-100 for 1 h at room temperature. Primary antibodies, including TC1HDG, phosphorylation-independent TDP-43 antibody (1:200, rabbit polyclonal; no. 12782-1-AP, Proteintech) and phosphorylation-dependent TDP-43 antibody (1:200, rat, clone 1D3; no. 829901, BioLegend) were applied and incubated overnight at 4 °C. Following washing with PBS, secondary antibodies were applied for 1 h at room temperature: Alexa Fluor 488 anti-rabbit IgG (1:400; no. ab150077, abcam), Alexa Fluor 568 anti-mouse IgG (1:400; no. ab175701, abcam) and Alexa Fluor 647 anti-rat IgG (1:400; no. ab150159, abcam), along with DAPI (1:1,000; catalog no. 10236276001, Roche). Following washing with PBS, sections were coverslipped using ProLong Gold Antifade Mountant (no. P36930, Invitrogen). Slides were examined on a Leica Mica confocal microscope.

### Biofluid selection

Biofluid samples were acquired from multiple cohorts. Feasible sample donation number and volume were determined in collaboration with biorepository leaders, and as many samples as possible were assayed. Neither sex nor gender was factored into sample selection to prioritize the number of samples acquired, but statistical analyses were performed to evaluate differences in cryptic HDGFL2 levels between males and females.

### CSF samples

Cerebrospinal fluid samples from *C9orf72* mutation carriers were provided by the Natural History and Biomarker Study of *C9orf72* ALS (protocol no. 13-N-0188) with enrollment/study period 2013–2020 (ref. ^21^) (NINDS), the DIALS Network and the NEALS Biorepository. CSF samples from sporadic ALS subjects were provided by the NEALS Biorepository and Biogen, Inc./PrecisionMed. Control CSF samples were provided by DIALS and the Johns Hopkins Bayview Medical Center. DIALS control samples were obtained from healthy controls. Bayview control samples were obtained from two cohorts: individuals with NPH and those with migraine.

Phosphorylated neurofilament heavy chain levels in CSF were measured by Biogen using the ProteinSimple Ella microfluidic immunoassay according to the manufacturer’s instructions. NfL levels in CSF were measured by Biogen using the Quanterix Simoa assay.

### Plasma samples

Plasma samples from *C9orf72* mutation carriers were provided by the Natural History and Biomarker Study of *C9orf72* ALS (protocol no. 13-N-0188) with enrollment/study period 2013–2020 (ref. ^21^). Disease control plasma samples were obtained from the same two Johns Hopkins Bayview Medical Center cohorts as the disease control CSF samples: individuals with NPH and those with migraine.

### MSD ELISA

Meso Scale Discovery assays were conducted on MSD MULTI-ARRAY 96-well SECTOR plates. For our novel sandwich ELISA using the MSD platform, our novel antibody (TC1HDG) against the cryptic exon-encoded peptide target in HDGFL2 served as the capture antibody. Plates were coated at 4 °C overnight with 40 μl per well of capture antibody at a concentration of 5 μg ml^−1^ in PBS. The following day, plates were washed with 150 μl of PBS-0.05% Tween (PBS-T) and blocked with 150 μl of 5% bovine serum albumin (BSA) in PBS-T for 1 h at room temperature, with shaking at 700 r.p.m. Plates were washed with PBS-T, and the standard curve and CSF/plasma samples were added.

Cerebrospinal fluid samples were assayed in duplicates of 50 μl diluted in 50 μl of 1% BSA in PBS, for a total of 100 μl added per well. Plasma samples were assayed in duplicates of 50 μl diluted in 50 μl of MSD diluent 57. The standard curve was constructed using a concentration series of purified cryptic HDGFL2 (Extended Data Fig. 2). This curve was employed in duplicates on each plate to normalize signals based on a four-parameter logistic fit. Purified cryptic HDGFL2 was diluted in 1% BSA in PBS for CSF assays and in diluent 57 for plasma assays.

Duplicates were placed on opposite sides of the MSD plate about both *x* and *y* axes. Eleven of the 44 sporadic ALS CSF samples did not have a duplicate due to volume restrictions. Samples were incubated for 2 h at room temperature, with shaking at 700 r.p.m.

In longitudinal assays, all time points of CSF collection for one individual were assayed on the same plate. Immediately before use, CSF or plasma was thawed, vortexed well and briefly spun down. All CSF samples had not previously been thawed or had undergone one previous freeze–thaw cycle. All CSF from both the DIALS cohort and disease controls had never been thawed, and all CSF from the NINDS and Biogen cohorts had undergone one previous freeze–thaw cycle. Of the NEALS *C9orf72* mutation carrier cohort, four CSF samples had previously been thawed once while the other 13 had never been thawed. Seventeen sporadic ALS CSF samples had been previously thawed once before MSD analysis while 27 had never been thawed. Plasma samples were obtained from the NINDS and Bayview disease control cohorts. Those from Bayview had not previously been thawed, while six NINDS plasma samples had previously been thawed once. For comparison of all samples together (Figs. 5 and 6), any samples previously thawed once were normalized based on cryptic HDGFL2 signal differences between a previously thawed aliquot and a never-previously thawed aliquot of a set of CSF (Extended Data Fig. 4) or plasma samples (Extended Data Fig. 8). All DIALS samples were analyzed in a blinded fashion.

A polyclonal goat antibody (gTEA1.2) against the C terminus of the WT protein was used as the detection antibody. The MSD GOLD SULFO-TAG NHS-Ester kit was used to conjugate this goat antibody with a sulfo-tag required for generation of electrochemiluminescent signal. The antibody was sulfo-tagged at a challenge ratio of 20:1 for a sulfo-tag label:protein conjugation ratio of 10.87:1. This antibody was diluted in MSD diluent 100, and 25 μl was added per well at a concentration of 5 μg ml^−1^ following biofluid incubation. The detection antibody was incubated for 2 h at room temperature, with shaking at 700 r.p.m.

The goat IgG isotype control antibody used for MSD testing (Fig. 4e) was sulfo-tagged at a challenge ratio identical to that of gTEA1.2 (20:1), for a conjugation ratio of 9.19:1.

Previously, a commercial antibody against the WT protein was used as the primary detection antibody (Anti-CTB-50L17.10 antibody produced in rabbit; Prestige Antibodies Powered by Atlas Antibodies), and a species-specific sulfo-tagged antibody (Anti Rabbit Antibody Goat SULFO-TAG Labeled; MSD) was used as a secondary detection reagent to generate electrochemiluminescence (Extended Data Fig. 6).

Following incubation of the detection antibody, plates were washed with PBS-T and 150 μl of MSD GOLD Read Buffer A was added to each well. Plates were immediately measured on the MESO QuickPlex SQ 120 MM instrument.

The LoD for CSF and plasma cryptic HDGFL2 was estimated by measuring 20 wells of diluent only with the MSD assay and calculating each signal as a ratio over the mean signal for the 20 wells. The LoD was then calculated as the mean ratio plus two standard deviations^59^. Following normalization of CSF and plasma signals to the diluent-only signal, cryptic HDGFL2 concentrations were considered ‘detectable’ only if CSF/plasma samples had signal ratios above the LoD. For cryptic HDGFL2 concentration comparisons between groups, signal ratios below the LoD were converted to cryptic HDGFL2 concentrations of 0 ng ml^−1^.

The intra-assay CV was calculated from 78 CSF samples assayed in duplicate on two full MSD plates (39 duplicates on each of two plates). All 78 samples came from the NINDS cohort and had previously been thawed once. Duplicates were oriented on opposite ends of the MSD ELISA plate about both *x* and *y* axes. Inter-assay CV calculation was performed using ten CSF samples from different individuals in the DIALS cohort because excess CSF aliquots were available for these subjects. For these ten cases, two different aliquots of CSF collected at the same time were assayed 7–8 weeks apart. MSD values were normalized to the diluent-only signal on each plate, and these ratios were used for inter-assay CV calculation.

### Statistics and reproducibility

Statistical analyses were performed in STATA v.17 (StataCorp) and GraphPad Prism v.9.4.1. Because of the highly skewed distribution of cryptic HDGFL2 MSD signals, analyses were performed using nonparametric tests unless otherwise stated. Correlation analyses were performed with the Spearman correlation unless otherwise stated. Group comparisons were performed using the Mann–Whitney *U*-test for continuous variables and Fisher’s exact test for categorical variables, and the Holm–Bonferroni correction was applied for adjustment of *P* values of multiple comparisons. For Mann–Whitney *U*-tests and Fisher’s exact tests comparing cryptic HDGFL2 levels between groups, cryptic HDGFL2 signal ratios or concentrations measured from longitudinal samples were averaged to produce one data point per individual, maintaining independence of observations. *P* < 0.05 was considered statistically significant.

Reproducibility was noted through the repeating of multiple experiments. Antibody testing was repeated several times, with similar results. Following initial testing of candidate cryptic HDGFL2 monoclonal antibody lines using GFP–myc-cryptic peptide fusion proteins (Supplementary Fig. 2), the no. 1-69 cryptic antibody line (TC1HDG) and the WT HDGFL2 antibody were tested on TDP-43 knockdown and control cell lysates (Fig. 2c) over ten times. IP blot using the TC1HDG antibody for pulldown and the WT HDGFL2 antibody for blotting (Fig. 2d) was repeated using these lysates over five times. TDP-43 knockdown by siRNA was validated by immunoblot (Fig. 2a) over ten times, and the *HDGFL2* cryptic exon was identified in TDP-43 knockdown cells by PCR and gel electrophoresis (Fig. 2b) over five times.

Reproducibility of TC1HDG immunostaining was also evaluated. Similar findings (Fig. 3) were identified in at least nine other ALS–FTD cases.

Wild-type and cryptic HDGFL2 overexpression lysates used for MSD development were also validated several times. Expected cryptic and WT HDGFL2 bands (Fig. 4b,c) were identified by immunoblot at least twice. MSD specificity for cryptic HDGFL2 was evaluated using these WT and cryptic HDGFL2 overexpression lysates (Fig. 4d) at least five times. Purified cryptic HDGFL2 used for the MSD standard curve was validated twice by immunoblot (Extended Data Fig. 2a).

Biofluid samples were acquired from multiple cohorts to determine reproducibility. CSF samples from *C9orf72* mutation carriers were acquired from three different cohorts, and sporadic ALS CSF samples were acquired from two different cohorts. MSD findings were reproducible across cohorts.

### Reporting summary

Further information on research design is available in the Nature Portfolio Reporting Summary linked to this article.

## Online content

Any methods, additional references, Nature Portfolio reporting summaries, source data, extended data, supplementary information, acknowledgements, peer review information; details of author contributions and competing interests; and statements of data and code availability are available at 10.1038/s41591-023-02788-5.

### Supplementary information

Supplementary Information Supplementary Figs. 1–3 and Table 1.

Reporting Summary

Supplementary Data 1 Participant demographic, clinical and biomarker data.

### Source data

Source Data Fig. 1 Unprocessed immunoblots and gels.

## Figures and Tables

**Extended Data Table 1:**
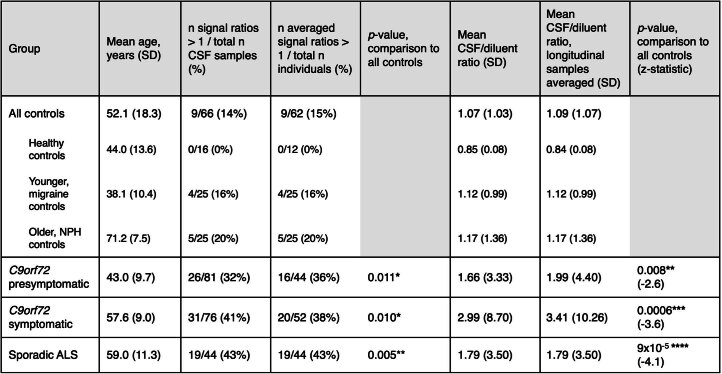
CSF cryptic HDGFL2 levels are significantly higher in sporadic ALS and presymptomatic and symptomatic *C9orf72* mutation carriers compared to controls

Statistical analyses were performed with longitudinal measurements averaged so that each individual contributed one data point. Frequencies of CSF/diluent-only signal ratios > 1 were compared between disease groups and all controls with Fisher’s exact test (two-tailed). Signal ratios were compared between disease groups and all controls with the Mann-Whitney U test (two-tailed). The Holm-Bonferroni correction was applied to all *p*-values. *: p ≤ 0.05, **: p ≤ 0.01, ***: p ≤ 0.001, ****: p ≤ 0.0001.

## Data Availability

Analyzed RNA sequencing data are available on NCBI’s Sequence Read Archive under SRA study nos. SRP166282 (ref. ^11^) and SRP057819 (ref. ^7^). NAUC data tables are available at http://ascot.cs.jhu.edu/ (ref. ^19^). The WT HDGFL2 protein structure can be found on the AlphaFold protein structure database (UniProt: Q7Z4V5). Relevant biomarker, demographic and clinical data associated with the biofluid samples analyzed here are available in the Supplementary Data Excel file. Full scans of gels and blots are available in Source Data. Novel monoclonal antibody against cryptic HDGFL2 and gTEA1.2 antibody against WT HDGFL2 are available for sharing from the laboratory of P.C.W. by request. All other antibodies are commercially available. Source Data are provided with this paper.
