# Author Correction: A fluid biomarker reveals loss of TDP-43 splicing repression in presymptomatic ALS–FTD

**DOI:** 10.1038/s41591-024-02966-z

**Published:** 2024-04-05

**Authors:** Katherine E. Irwin, Pei Jasin, Kerstin E. Braunstein, Irika R. Sinha, Mark A. Garret, Kyra D. Bowden, Koping Chang, Juan C. Troncoso, Abhay Moghekar, Esther S. Oh, Denitza Raitcheva, Dan Bartlett, Timothy Miller, James D. Berry, Bryan J. Traynor, Jonathan P. Ling, Philip C. Wong

**Affiliations:** 1 Department of Pathology, Johns Hopkins Medicine, Baltimore, MD USA; 2 Department of Neuroscience, Johns Hopkins Medicine, Baltimore, MD USA; 3 Sean M. Healey & AMG Center for ALS, Massachusetts General Hospital, Boston, MA USA; 4 Department and Graduate Institute of Pathology, National Taiwan University Hospital, National Taiwan University College of Medicine, Taipei, Taiwan; 5 Department of Neurology, Johns Hopkins Medicine, Baltimore, MD USA; 6 Department of Medicine, Johns Hopkins Medicine, Baltimore, MD USA; 7 Department of Psychiatry and Behavioral Sciences, Johns Hopkins Medicine, Baltimore, MD USA; 8 Biogen, Cambridge, MA USA; 9 Department of Neurology, Washington University School of Medicine in St. Louis, St. Louis, MO USA; 10 Neuromuscular Diseases Research Section, National Institute on Aging, National Institutes of Health, Bethesda, MD USA; 11 National Institute of Neurological Disorders, National Institutes of Health, Bethesda, MD USA; 12 RNA Therapeutics Laboratory, Therapeutics Development Branch, National Center for Advancing Translational Sciences, National Institutes of Health, Rockville, MD USA

**Keywords:** Amyotrophic lateral sclerosis, Diagnostic markers

Correction to: *Nature Medicine* 10.1038/s41591-023-02788-5, published online 26 January 2024.

In the version of the article initially published, in the “Analysis with reverse transcription PCR” section of the Methods, the HDG F primer originally read 5′-CTCGGAGGCTTCTTCACAGAC-3′ and has now been corrected to 5′-AAGACGCCTGCGCTAAAGAT-3′ in the HTML and PDF versions of the article.

