# Supplementary material for: A fluid biomarker reveals loss of TDP-43 splicing repression in presymptomatic ALS–FTD
Source: Nat Med. 2024 Jan 26;30(2):382–93. doi: 10.1038/s41591-023-02788-5 (PMC10878965; doi:10.1038/s41591-023-02788-5)
Supplement: Supplementary file 1 — Supplementary Figs. 1–3 and Table 1. [file 41591_2023_2788_MOESM1_ESM.pdf]

nature medicine

Article https://doi.org/10.1038/s41591-023-02788-5

A fluid biomarker reveals loss of TDP-43 splicing repression in presymptomatic ALS–FTD

In the format provided by the authors and unedited

Supplementary Figures and Tables

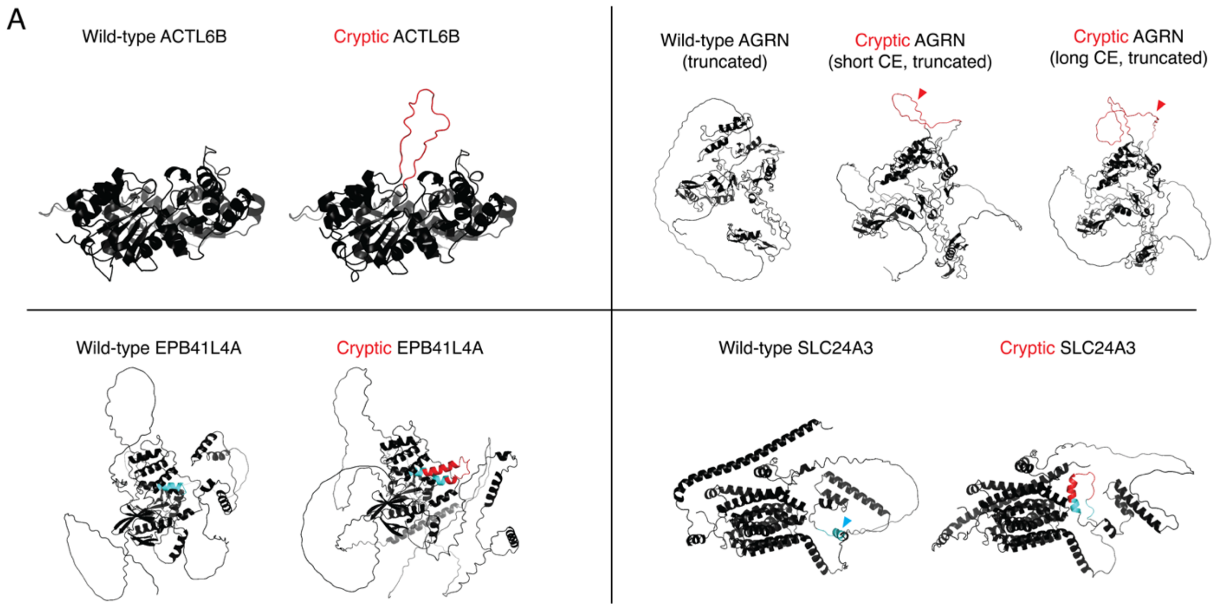

B

```
Wild-type ACTL6B   109 KSEPNLHPVLMSEAP-----------------------WNTRAKREKLTELMF 138
Cryptic ACTL6B     109 KSEPNLHPVLMSEAPASFELLGSSNPPASASQSAGIIGWNTRAKREKLTELMF 161

Wild-type AGRN     738 SQRGLYVAAQGACRG--------------------------------PTFAPLPPVAPLHC 766
Cryptic AGRN       738 SQRGLYVAAQGACRARVCVCTELRVRDLWMLCVLCVPGSAFTDMFLRPTFAPLPPVAPLHC 798

Wild-type AGRN     738 SQRGLYVAAQGACRG---------------------------------------------------------------PTFAPLPPVAPLHC 766
Cryptic AGRN       738 SQRGLYVAAQGACRARVCVCTELRVRDLWMLCVLCVPGSAFTDMFLRKMWEPHLCPQPQAPGLLWELGSGRLSGDSGGPTFAPLPPVAPLHC 829

Wild-type EPB41L4A 98  FYAEDPCKLKEEITR-------------------------YQFFLQVKQDVLQG 126
Cryptic EPB41L4A   98  FYAEDPCKLKEEITSDIESPYKTEVTKGQAEVCESVCAYVYQFFLQVKQDVLQG 151

Wild-type SLC24A3  286 DDSSNCDATVVLLKK----------------ANFHRKASVIMVDEL 313
Cryptic SLC24A3    286 DDSSNCDATVVLLKKGRKEGIRKEEWMDGWIANFHRKASVIMVDEL 331
```

Supplementary Fig. 1: Visualization of additional human in-frame TDP-43-associated cryptic exons. (**A**) Comparison of wild-type and cryptic ACTL6B (top left), AGRN (top right), EPB41L4A (bottom left), and SLC24A3 (bottom right) protein structures. Cryptic exons are indicated in red and match the amino acid sequences in **B**. Red arrows in cryptic AGRN indicate the location of the cryptic exon, and the cyan arrow for wild-type SLC24A3 points towards the flanking amino acids to aid identification. Full wild-type protein structures can be found on the AlphaFold protein structure database: ACTL6B (UniProt: O94805), EPB41L4A (UniProt: Q9HCS5), and SLC24A3 (UniProt: Q9HC58). Due to the size of the AGRN protein, a truncated version was generated using AlphaFold. (**B**) Alignment of wild-type and cryptic ACTL6B, AGRN, EPB41L4A, and SLC24A3 amino acid sequences. Cryptic inclusion is in red for all proteins, and the flanking amino acids are visualized in cyan for EPB41L4A and SLC24A3.

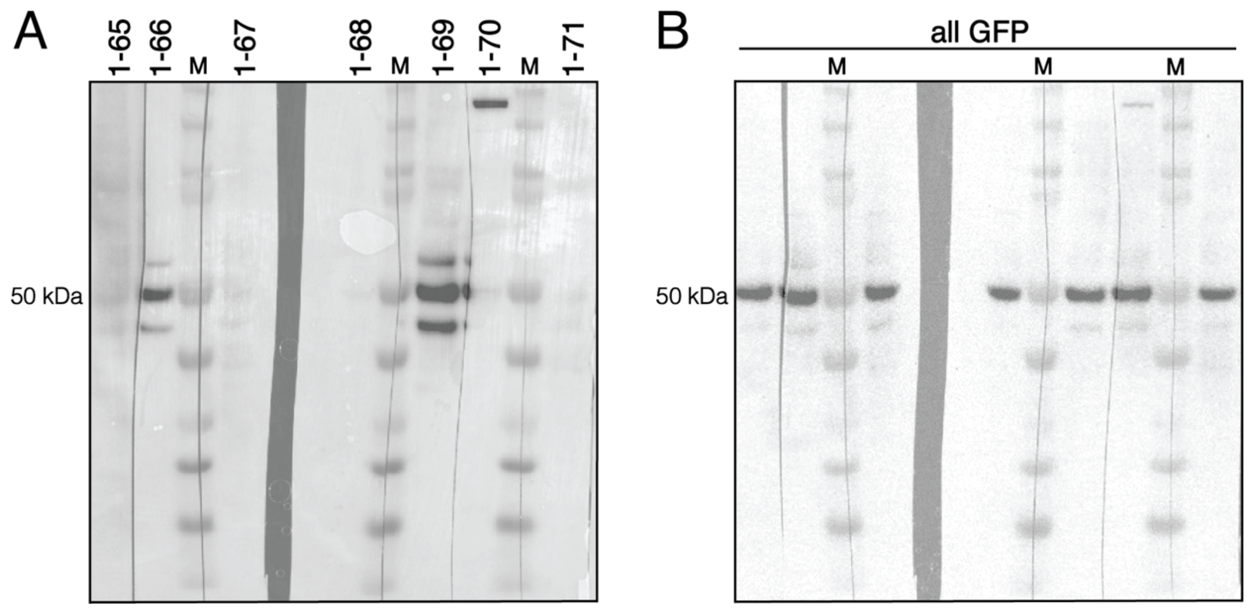

Supplementary Fig. 2: Generation of a series of antibodies recognizing a cryptic peptide in HDGFL2. (**A**) HEK293 cells were transfected with a plasmid expressing a fusion protein of GFP, a myc tag, and the cryptic peptide within HDGFL2. Lysates were subjected to protein blot analysis using antisera from 7 different monoclonal lines against cryptic HDGFL2. Lines 1-66 and 1-69 detected the fusion protein containing the cryptic HDGFL2 peptide. (**B**) Immunoblots of the same lysates using anti-GFP antisera confirmed the identity of the GFP-myc-cryptic HDGFL2 fusion protein. M: molecular weight.

Neuropathologic diagnosis and brain region	Age (years)	Sex	Race
ALS/FTLD-TDP motor cortex	63	Female	White
Normal motor cortex	38	Male	White
C9orf72 FTLD-TDP hippocampus	67	Male	White
Normal hippocampus	27	Male	White

Supplementary Table 1: Participant data for Figure 3 brain samples.

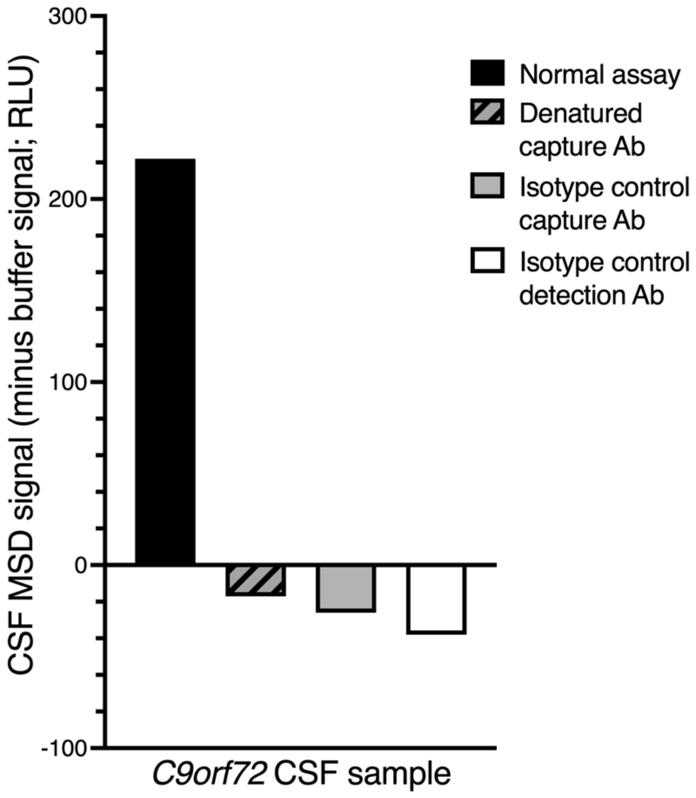

Supplementary Fig. 3: Development of an MSD assay specific for cryptic HDGFL2. Elevated MSD signal of *C9orf72* CSF sample is specific to intact capture and detection antibodies. Denatured capture antibody was heated at 95°C for 30 minutes. Mouse IgG and sulfo-tagged goat IgG were used as isotype controls for capture and detection antibodies, respectively. RLU: relative light units.
