# Supplementary material for: A fluid biomarker reveals loss of TDP-43 splicing repression in presymptomatic ALS–FTD
Source: Nat Med. 2024 Jan 26;30(2):382–93. doi: 10.1038/s41591-023-02788-5 (PMC10878965; doi:10.1038/s41591-023-02788-5)
Supplement: Supplementary file 2 — Reporting Summary [file 41591_2023_2788_MOESM2_ESM.pdf]

nature portfolio

Corresponding author(s): Philip C. Wong

Last updated by author(s): Jan 12, 2024

# Reporting Summary

Nature Portfolio wishes to improve the reproducibility of the work that we publish. This form provides structure for consistency and transparency in reporting. For further information on Nature Portfolio policies, see our Editorial Policies and the Editorial Policy Checklist.

## Statistics

For all statistical analyses, confirm that the following items are present in the figure legend, table legend, main text, or Methods section.

| n/a | Confirmed | |
|---|---|---|
| ☐ | ☒ | The exact sample size (*n*) for each experimental group/condition, given as a discrete number and unit of measurement |
| ☐ | ☒ | A statement on whether measurements were taken from distinct samples or whether the same sample was measured repeatedly |
| ☐ | ☒ | The statistical test(s) used AND whether they are one- or two-sided<br>*Only common tests should be described solely by name; describe more complex techniques in the Methods section.* |
| ☒ | ☐ | A description of all covariates tested |
| ☐ | ☒ | A description of any assumptions or corrections, such as tests of normality and adjustment for multiple comparisons |
| ☐ | ☒ | A full description of the statistical parameters including central tendency (e.g. means) or other basic estimates (e.g. regression coefficient) AND variation (e.g. standard deviation) or associated estimates of uncertainty (e.g. confidence intervals) |
| ☐ | ☒ | For null hypothesis testing, the test statistic (e.g. *F*, *t*, *r*) with confidence intervals, effect sizes, degrees of freedom and *P* value noted<br>*Give P values as exact values whenever suitable.* |
| ☒ | ☐ | For Bayesian analysis, information on the choice of priors and Markov chain Monte Carlo settings |
| ☒ | ☐ | For hierarchical and complex designs, identification of the appropriate level for tests and full reporting of outcomes |
| ☐ | ☒ | Estimates of effect sizes (e.g. Cohen's *d*, Pearson's *r*), indicating how they were calculated |

*Our web collection on statistics for biologists contains articles on many of the points above.*

## Software and code

Policy information about availability of computer code

| | |
|---|---|
| Data collection | No software was used for data collection in this study. |
| Data analysis | For alignment and visualization of RNA-seq data: SRA tools (sra-tools/3.0.0), STAR (2.7.10a), megadepth (1.5.0), UCSC Genome Browser (http://genome.ucsc.edu).<br>For visualization of expression data: ASCOT, R (4.1.1), ggplot2 package in R (3.4.0)<br>Protein structure prediction and visualization: AlphaFold Monomer v2.0 pipeline (2.2.0), Benchling, Pymol (2.5.4).<br>Generally: Anaconda (2020.07), Python (3.6.8). |

For manuscripts utilizing custom algorithms or software that are central to the research but not yet described in published literature, software must be made available to editors and reviewers. We strongly encourage code deposition in a community repository (e.g. GitHub). See the Nature Portfolio guidelines for submitting code & software for further information.

## Data

Policy information about availability of data

All manuscripts must include a data availability statement. This statement should provide the following information, where applicable:

- Accession codes, unique identifiers, or web links for publicly available datasets
- A description of any restrictions on data availability
- For clinical datasets or third party data, please ensure that the statement adheres to our policy

RNA-seq data analyzed is available on NCBI's Sequence Read Archive under SRA study numbers SRP166282 (Klim et al., 2019) and SRP057819 (Ling et al., 2015).
NAUC data tables are available on http://ascot.cs.jhu.edu/ (Ling et al., 2020).
Wild-type HDGFL2 protein structure can be found on the AlphaFold protein structure database (UniProt: Q7Z4V5).

## Human research participants

Policy information about studies involving human research participants and Sex and Gender in Research.

| | |
|---|---|
| Reporting on sex and gender | Information on sex of participants is included in a data table uploaded as Supplementary Data |
| Population characteristics | Population characteristics are described in the manuscript and are included in a data table uploaded as Supplementary Data |
| Recruitment | CSF samples were collected by other groups according to previously published protocols. This study did not directly involve human research participants. Participant recruitment for NINDS C9orf72 mutation carriers is detailed in protocol #13-N-0188 for clinical trial NCT01925196. |
| Ethics oversight | All brains were examined in the Division of Neuropathology at Johns Hopkins under a protocol approved by the JHU IRB. |

Note that full information on the approval of the study protocol must also be provided in the manuscript.

# Field-specific reporting

Please select the one below that is the best fit for your research. If you are not sure, read the appropriate sections before making your selection.

☒ Life sciences ☐ Behavioural & social sciences ☐ Ecological, evolutionary & environmental sciences

For a reference copy of the document with all sections, see nature.com/documents/nr-reporting-summary-flat.pdf

# Life sciences study design

All studies must disclose on these points even when the disclosure is negative.

| | |
|---|---|
| Sample size | Samples sizes were not predetermined. As many control, C9orf72 mutation carrier, and sporadic ALS samples were acquired as was feasible given the precious nature of such samples. With 66 control, 81 presymptomatic C9, 76 symptomatic C9, and 44 sporadic ALS CSF samples, we were able to show statistical significance between disease groups and controls. |
| Data exclusions | One CSF cryptic HDGFL2 MSD signal was out of range of our four parameter logistic curve normalization and was excluded. This information is included in the Supplementary Data table. |
| Replication | Antibody testing was repeated several times with similar results. Following initial testing of candidate cryptic HDGFL2 monoclonal antibody lines using GFP-myc-cryptic peptide fusion proteins (Supplementary Fig. 2), the #1-69 cryptic antibody line (TC1HDG) and the wild-type HDGFL2 antibody were tested on TDP-43 knockdown and control cell lysates (Fig. 2C) over ten times. IP blot using the TC1HDG antibody for pull-down and the WT HDGFL2 antibody for blotting (Fig. 2D) was repeated using these lysates over five times. The TDP-43 knockdown by siRNA was validated by Western blot (Fig. 2A) over ten times, and the HDGFL2 cryptic exon was identified in TDP-43 knockdown cells by PCR and gel electrophoresis (Fig. 2B) over five times.<br>Reproducibility of TC1HDG immunostaining was also evaluated. Similar findings (Fig. 3) were identified in at least 9 other ALS-FTD cases.<br>WT and cryptic HDGFL2 overexpression lysates used for MSD development were also validated several times. Expected cryptic and WT HDGFL2 bands (Fig. 4B, C) were identified by Western blot at least twice. MSD specificity for cryptic HDGFL2 was evaluated using these WT and cryptic HDGFL2 overexpression lysates (Fig. 4D) at least five times. Purified cryptic HDGFL2 used for the MSD standard curve was validated by Western blot (Extended Data Fig. 2A) twice. |
| Randomization | Randomization of experimental groups is not relevant to this study. Human subjects were identified as controls, presymptomatic C9orf72 mutation carriers, symptomatic C9orf72 mutation carriers, or sporadic ALS patients based on genetic data and clinical diagnosis. Samples were measured as each cohort became available. Blinding (see below) was used to prevent bias within cohorts. |
| Blinding | CSF samples were labeled with subject ID number and did not display information related to patient diagnosis. The DIALS cohort was analyzed in a blinded fashion. For the other cohorts, the patient data were not blinded, but this information was not viewed during the experimental assays. Western blot experiments did not warrant blinding. Immunostaining of patient brain was not blinded but was repeated several times. |

# Reporting for specific materials, systems and methods

We require information from authors about some types of materials, experimental systems and methods used in many studies. Here, indicate whether each material, system or method listed is relevant to your study. If you are not sure if a list item applies to your research, read the appropriate section before selecting a response.

## Materials & experimental systems

| n/a | Involved in the study |
| --- | --- |
| ☐ | ☒ Antibodies |
| ☐ | ☒ Eukaryotic cell lines |
| ☒ | ☐ Palaeontology and archaeology |
| ☒ | ☐ Animals and other organisms |
| ☐ | ☒ Clinical data |
| ☒ | ☐ Dual use research of concern |

## Methods

| n/a | Involved in the study |
| --- | --- |
| ☒ | ☐ ChIP-seq |
| ☒ | ☐ Flow cytometry |
| ☒ | ☐ MRI-based neuroimaging |

## Antibodies

Antibodies used

The following antibodies were used for Western blot:
Wild-type HDGFL2 - HPA044208;
TDP-43 - 10782-2-AP;
GAPDH - D16H11 XP® Rabbit mAb #5174;
Novel monoclonal antibody against cryptic HDGFL2 (#1-69, TC1HDG) - purified from hybridomas generated by CDI Laboratories Inc.;
Custom goat antibody against wild-type HDGFL2 (gTEA1.2) - Rockland Immunochemicals;
Goat anti-mouse IgG HRP - 32230;
Goat anti-rabbit IgG HRP - BP-9100-50;
Bovine anti-goat IgG HRP - 805-035-180.

The following antibodies were used for immunostaining:
Novel monoclonal antibody against cryptic HDGFL2 (TC1HDG) - purified from hybridomas generated by CDI Laboratories Inc.;
TDP-43 - 10782-2-AP;
phospho-TDP-43 - 829901;
Rb 488 - ab150077;
Ms 568 - ab175701;
Rat 647 - ab150167.

The following antibodies were used for MSD ELISA:
Wild-type HDGFL2 - HPA044208;
Rabbit IgG (sulfo-tagged) - Meso Scale Discovery Anti Rabbit Antibody Goat SULFO-TAG Labeled, R32AB-1, lot W0020326S;
Novel monoclonal antibody against cryptic HDGFL2 (TC1HDG) - purified from hybridomas generated by CDI Laboratories Inc.;
Custom goat antibody against wild-type HDGFL2 (gTEA1.2) - Rockland Immunochemicals.

Validation

Wild-type HDGFL2 - validated by the supplier with the following notes: Validation of protein expression in IHC by comparing independent antibodies targeting different epitopes of the protein. Validated against independent antibody Anti-CTB-50L17.10 HPA042559.
TDP-43 - validated by the supplier with the following notes: species reactivity - human; application - Western blot, IHC, IF.
phospho-TDP-43 - validated by the supplier with the following notes: species reactivity - human; application - Western blot; IHC, IF reported in literature.
GAPDH - validated by the supplier with the following notes: species reactivity - human; application - Western blot
Novel monoclonal antibody against cryptic HDGFL2 (#1-69) - validated by study authors KEI, KEB, and PJ by Western blot
Goat antibody against wild-type HDGFL2 (gTEA1.2) - validated by study author KEI by Western blot

## Eukaryotic cell lines

Policy information about cell lines and Sex and Gender in Research

Cell line source(s)

HeLa cells were purchased from ATCC. HEK293 cells were purchased from Millipore Sigma (catalog number 96121229-1VL) and from ATCC.

Authentication

HeLa and HEK293 cells were not authenticated.

Mycoplasma contamination

HeLa and HEK293 cells were negative for mycoplasma contamination as tested by ATCC and ECACC. Cells were not tested for mycoplasma contamination after purchase.

Commonly misidentified lines
(See ICLAC register)

No commonly misidentified lines were used.

## Clinical data

Policy information about clinical studies

All manuscripts should comply with the ICMJE guidelines for publication of clinical research and a completed CONSORT checklist must be included with all submissions.

| | |
|---|---|
| Clinical trial registration | This study was not a clinical trial. However, NINDS CSF samples were used from trial NCT01925196. |
| Study protocol | NINDS samples were used from protocol 13-N-0188. |
| Data collection | NINDS samples were used from participants who had up to 4 in-person visits at the National Institutes of Health Clinical Center over 3 years. The study began in 2013. Other samples/associated clinical data were provided by DIALS, NEALS, and Biogen. |
| Outcomes | Clinical trial outcomes are not relevant to this study. |
