# Supplementary material for: A fluid biomarker reveals loss of TDP-43 splicing repression in presymptomatic ALS–FTD
Source: Nat Med. 2024 Jan 26;30(2):382–93. doi: 10.1038/s41591-023-02788-5 (PMC10878965; doi:10.1038/s41591-023-02788-5)
Supplement: Supplementary file 4 — Unprocessed immunoblots and gels. [file 41591_2023_2788_MOESM4_ESM.pdf]

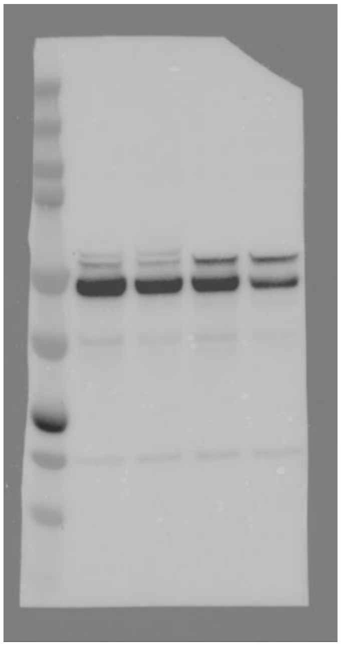
Figure 2A, upper

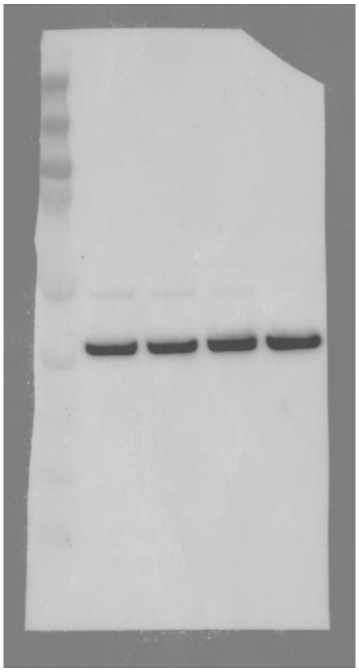
Figure 2A, lower

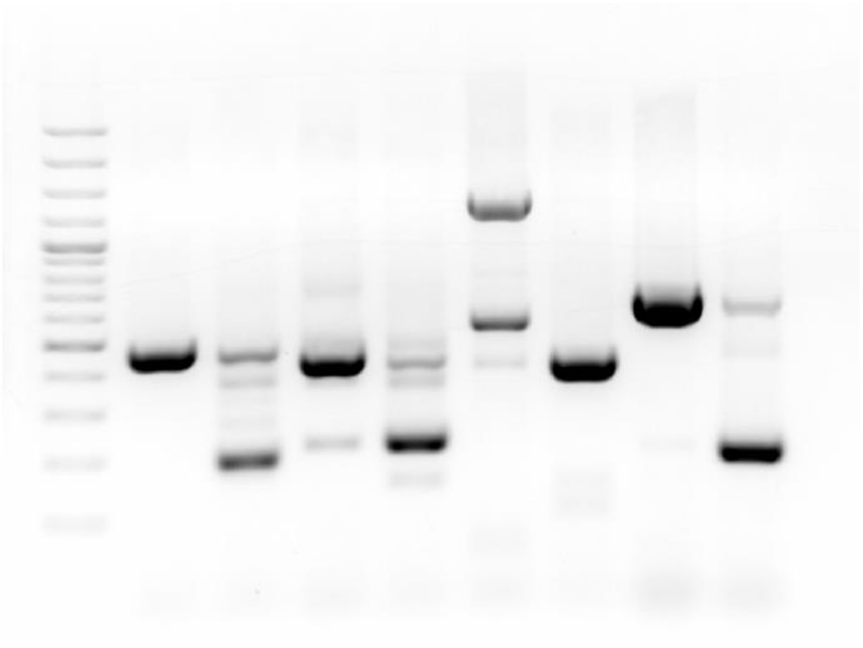

Figure 2B

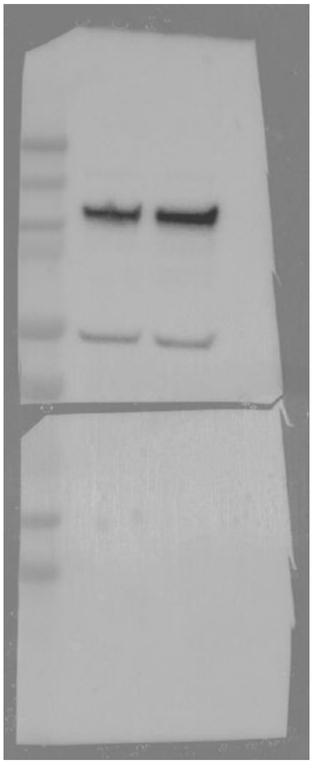
Figure 2C, left

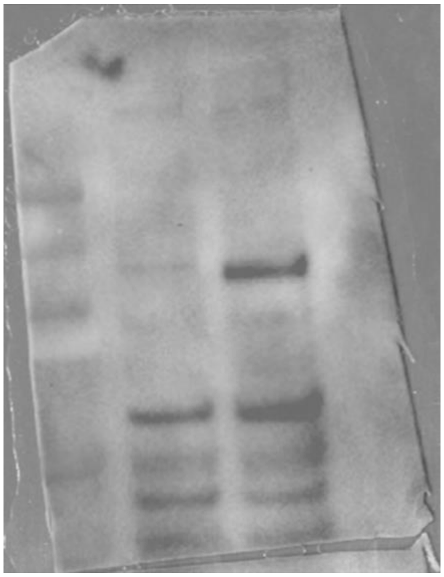
Figure 2C, right

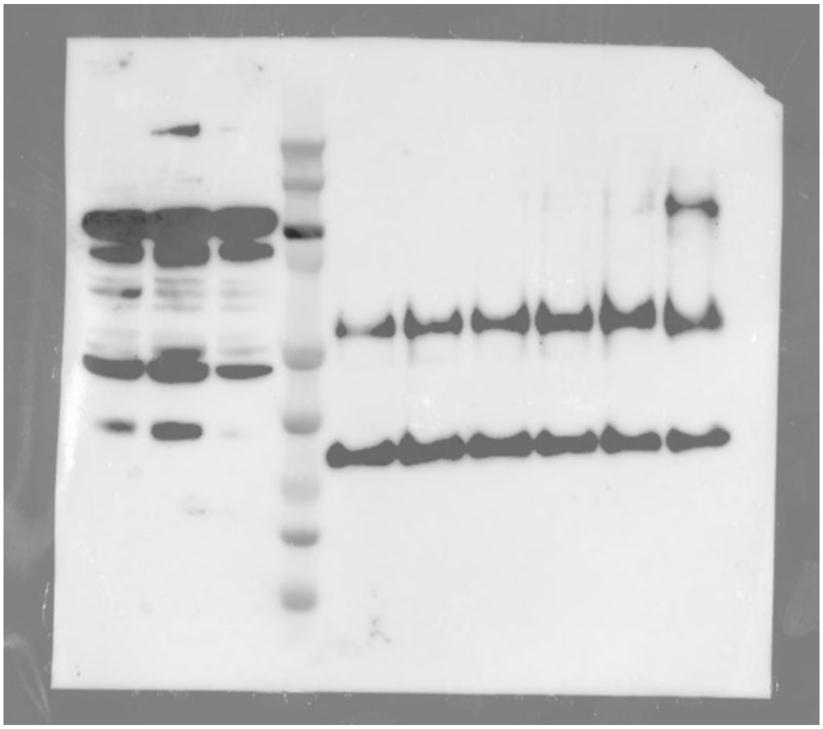

Figure 2D

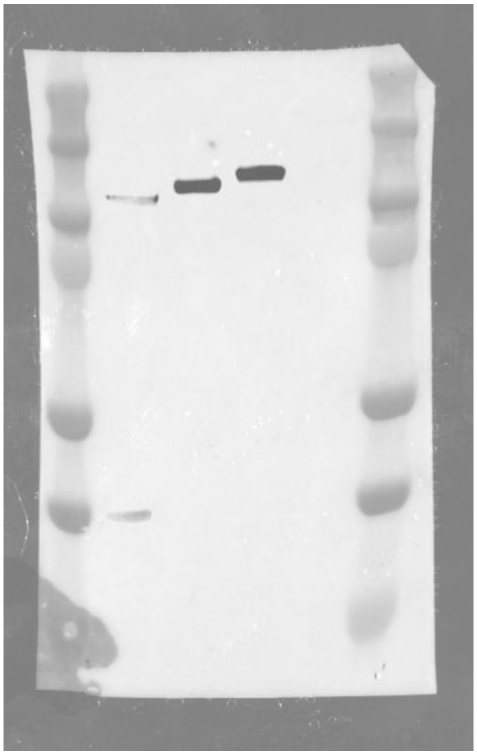
Figure 4B, upper

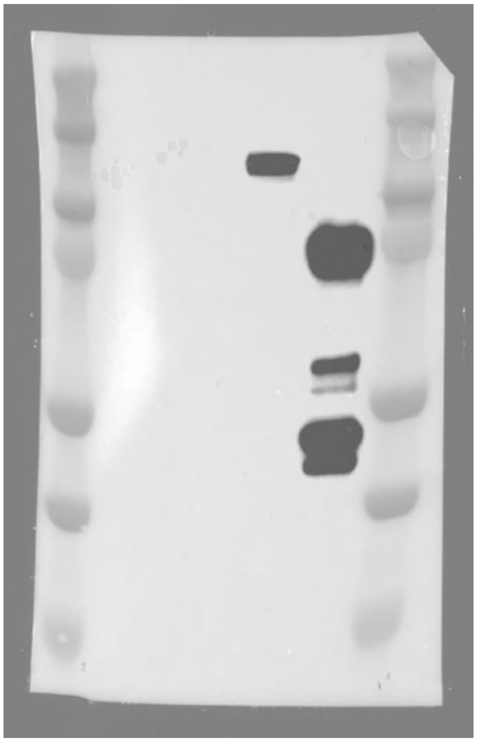
Figure 4B, lower

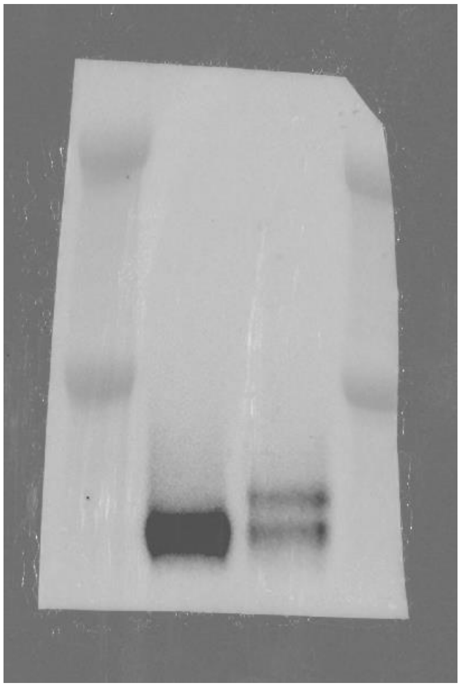
Figure 4C, upper

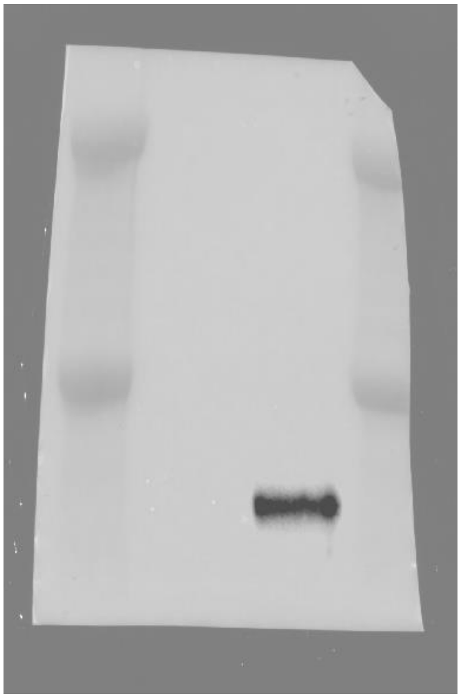
Figure 4C, lower

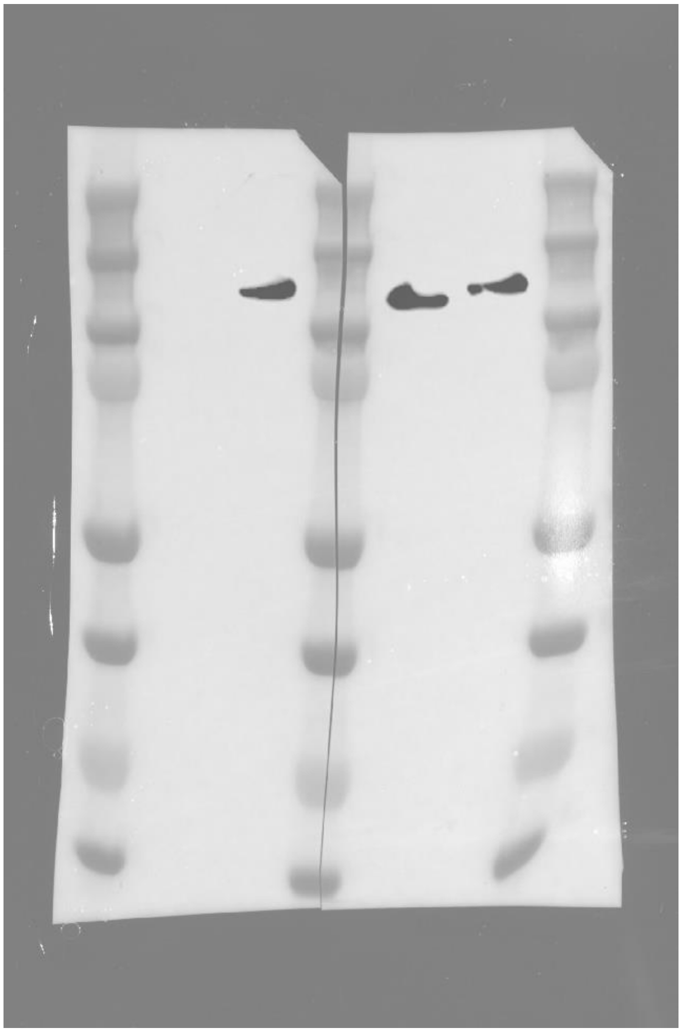

Extended Data Figure 2A
